# Targeted and Untargeted Metabolomics as an Enhanced Tool for the Detection of Pomegranate Juice Adulteration

**DOI:** 10.3390/foods8060212

**Published:** 2019-06-14

**Authors:** Marilena E. Dasenaki, Sofia K. Drakopoulou, Reza Aalizadeh, Nikolaos S. Thomaidis

**Affiliations:** Laboratory of Analytical Chemistry, Department of Chemistry, National and Kapodistrian University of Athens, Panepistimiopolis Zographou, 15771 Athens, Greece; sofiadrakop@chem.uoa.gr (S.K.D.); raalizadeh@chem.uoa.gr (R.A.); ntho@chem.uoa.gr (N.S.T.)

**Keywords:** fruit juice authenticity, pomegranate juice, adulteration, high-resolution mass spectrometry, biomarkers

## Abstract

Pomegranate juice is one of the most popular fruit juices, is well-known as a “superfood”, and plays an important role in healthy diets. Due to its constantly growing demand and high value, pomegranate juice is often targeted for adulteration, especially with cheaper substitutes such as apple and red grape juice. In the present study, the potential of applying a metabolomics approach to trace pomegranate juice adulteration was investigated. A novel methodology based on high-resolution mass spectrometric analysis was developed using targeted and untargeted screening strategies to discover potential biomarkers for the reliable detection of pomegranate juice adulteration from apple and red grape juice. Robust classification and prediction models were built with the use of unsupervised and supervised techniques (principal component analysis (PCA) and partial least squares discriminant analysis (PLS-DA)), which were able to distinguish pomegranate juice adulteration to a level down to 1%. Characteristic *m/z* markers were detected, indicating pomegranate juice adulteration, and several marker compounds were identified. The results obtained from this study clearly demonstrate that Mass Spectrometry (MS)-based metabolomics have the potential to be used as a reliable screening tool for the rapid determination of food adulteration.

## 1. Introduction

The fruit and vegetable juice industry is one of the world’s fastest-growing segments of the beverage industry due to the mounting focus of consumers on a healthy and balanced diet. As health and fitness have become vital in today’s world, changes in lifestyles have steered the growth of the global juice market across various developing and developed countries. According to the “Global Fruit and Vegetable Juice Market Research Report, 2018–2025”, the value of the global fruit juice market reached 154.18 billion USD in 2016 and is expected to grow at a compound annual growth rate (CAGR) of 5.93% during the forthcoming years [1]. 

Compared to other types of fruit juice, the popularity of pomegranate juice has skyrocketed in the last decade, mainly due to its well-established health benefits [2]. The pomegranate (*Punica granatum* L*.)* is an excellent source of precious nutrients, such as vitamins, sugars, acids, polysaccharides, polyphenols, and minerals, promoting an organism’s health and wellness. On top of that, pomegranate juice’s antioxidant activity has been repeatedly reported to be higher compared to that of other fruit juices [3]. Regular pomegranate juice consumption has been linked with the improvement of cardiovascular health through the reduction of cholesterol levels and the lowering of blood pressure, and also with the prevention of skin, breast, and prostate cancer [3,4,5]. With antimicrobial, anti-inflammatory, astringent, antitussive, and antidiarrheal properties, pomegranate juice has gained a reputation as an easily accessible superfood and is being sold as a high-quality food item [2]. 

Pomegranate juice’s economic value, along with its constantly increasing demand, which often exceeds supply, makes it vulnerable to adulteration [3]. The adulteration of pomegranate juice mostly includes dilution with water, the addition of sugars, or mixing with cheaper juices (apple, grape, pear) and is an illegal practice adopted by several suppliers and manufacturers to compensate for high product demand and mask low-quality raw materials [6]. This practice has a negative impact on the nutritional value of the juice and may also involve health risks for consumers, as the undeclared alterations in chemical composition could cause potential allergic effects [5]. Overall, adulteration reduces the product’s quality, deceiving consumers and violating their rights. Therefore, the monitoring of pomegranate juice authenticity by both regulatory agencies and the fruit juice industry is utterly essential [7]. 

In this context, the development of reliable, sensitive, and efficient analytical methodologies to detect pomegranate juice adulteration represents a demanding and challenging task. Conventional analytical techniques can be used to detect severe adulteration practices through the measurement of selected physicochemical indicators (pH, °Brix value, or titratable acidity), but they are often unable to detect small differences that could be indicative of low-level adulteration [8]. The most established approaches so far are based on targeted profiling of specific fruit juice constituents such as amino acids [2,9], polyphenols [4,10,11], and organic acids [2,6,12]. Based on this approach, Zhang et al. (2009) used a combination of existing databases and analytical techniques to characterize pure pomegranate juices and to establish authentication criteria by developing an international multidimensional authenticity specification (IMAS) algorithm [13]. Untargeted fingerprinting methodologies using spectroscopic techniques (Ultraviolet-visible spectroscopy, UV-VIS and Fourier-transform infrared spectroscopy, FTIR) combined with chemometrics have also been developed to unmask pomegranate juice adulteration through water addition or juice-to-juice adulteration with apple and grape juice [5,14]. 

In the last few years, a universal analytical approach called “metabolomics” has experienced a significant increase in interest in food fingerprinting studies [15,16]. Metabolomics focuses on the study of low-molecular-weight molecules (<1000 Da) and is used to explore and characterize food constituents, generating a detailed and comprehensive metabolic chemical profile of food. Metabolomic studies mainly involve the detection of metabolites (biomarkers) that can discriminate between sample populations (discriminative metabolomics) and/or the generation of statistical models able to classify samples and predict class memberships (predictive metabolomics) [17]. The identification and quantification of the biomarkers responsible for discrimination (informative metabolomics) is desirable but is not the main target in such studies [16]. In metabolomics, the use of high-throughput analytical techniques, such as high-resolution mass spectrometry (HRMS), is essential in order to enable the large-scale determination of unknown compounds. Statistical treatment using advanced chemometric tools, such as principal component analysis (PCA) and partial least squares discriminant analysis (PLS-DA), is necessary for the discrimination/classification of the samples and the development of predictive models [18].

Metabolomic studies have found, so far, limited application in fruit juice authenticity assessment, mainly concerning the detection of juice-to-juice adulteration of citrus fruits, while research regarding pomegranate juice adulteration is still scarce [8,19,20,21,22]. The main objective of this work was to evaluate the feasibility of targeted and untargeted Mass Spectrometry (MS)-based metabolomics, using ultraperformance liquid chromatography coupled to quadrupole time-of-flight mass spectrometry (UPLC-QTOF/MS), in the discrimination of authentic pomegranate juice and pomegranate juice adulterated with apple and red grape juice. The potential of this approach to detect low levels of juice-to-juice adulteration (down to 1%) was investigated, using both supervised (PLS-DA) and unsupervised (PCA) pattern recognition techniques. Finally, fingerprint compounds of apple and red grape juice were identified and structurally characterized and could be used as specific markers revealing pomegranate juice adulteration. 

## 2. Materials and Methods

### 2.1. Chemicals and Reagents

All standards and reagents used were of high-purity grade (>95%): 2,5-dihydroxybenzoic acid (gentistic acid), 3,4-dihydroxybenzoic acid (protocatechuic acid), 4-hydroxybenzoic acid, 8-prenylnaringenin, catechin, chrysin, cinnamic acid, gallic acid, ferulic acid, epicatechin, *p*-coumaric acid, quercetin, vanillic acid, pinoresinol, syringaldehyde, syringic acid, taxifolin, salicylic acid, rutin, rosmarinic acid, resveratrol, pinobanksin, pinocembrin, myricetin, and eriodictyol were obtained from Sigma-Aldrich (Stenheim, Germany). Luteolin, hydroxytyrosol, and 2ʹ,4ʹ-dihydroxychalcone were purchased from Santa Cruz Biotechnology (Santa Cruz, CA, USA), while tyrosol, caffeic acid, vanillin, ethyl vanillin, apigenin galangin, genistein, hesperetin, and naringenin were purchased from Alfa Aesar (Karlsruche, Germany).

Methanol (MeOH) (LC–MS grade) was purchased from Merck (Darmstadt, Germany), whereas 2-propanol (LC–MS grade) was purchased from Fisher Scientific (Geel, Belgium). Sodium hydroxide monohydrate for trace analysis ≥99.9995%, ammonium acetate, and formic acid 99% were purchased from Fluka (Buchs, Switzerland). Distilled water was provided by a Milli-Q purification apparatus (Millipore Direct-Q UV, Bedford, MA, USA). Finally, regenerated cellulose syringe filters (RC filters, pore size 0.2 μm, diameter 15 mm) were acquired from Phenomenex (Torrance, CA, USA). Stock standard solutions of individual compounds (1000 μg mL^−1^) were prepared in MeOH and stored at −20 °C in amber glass bottles to prevent photodegradation. Working mix solutions of concentrations from 0.25 to 10 mg/L for each analyte were prepared by gradient dilution of the stock solutions in methanol/water (50:50 *v/v*). 

### 2.2. Samples and Sample Preparation

Twenty-eight commercial, concentrated fruit juice samples (five 100% pomegranate juices, eight 100% apple juices, and 15 100% red grape juices), were directly supplied by a major Greek fruit juice company, DELTA FOODS S.A (Athens, Greece) (Table 1). With Turkey being an important and growing player in the pomegranate market, commercial pomegranate juices belonging to the Turkish Hicaz variety were selected for this study. Hicaz is the most produced and most consumed pomegranate variety in Turkey and is widely exported to European countries [23]. Apple juice samples included two apple cultivars (Starking and Granny Smith) from three different geographical regions of Greece (Western Macedonia, Central Macedonia, and Thessaly), and red grape juices consisted of pool samples mixing seven varieties (Sangiovese, Montepulciano, Lambrusco, Schiava, Shiraz, Ciliegiolo, and Merlot), which came from Italy (Puglia). All concentrated juice samples were produced in 2016 and were diluted to 11.2 ± 0.5 °Brix for apple juice, 15 ± 0.5 °Brix for pomegranate juice, and 15.9 ± 0.5 °Brix for red grape juice prior to analysis, according to the manufacturer’s instructions. Additionally, one freshly squeezed pomegranate juice was prepared from Ermioni variety fruits, hand-picked from an orchard in Argolida, Greece. The juice was prepared based on the sampling methodology of Arbona et al. [24]: At least eight fruits, two from each direction on the pomegranate tree, were collected from 10 replicate trees (*n* = 100), and their juice was extracted through manual squeezing. Commercial and freshly squeezed fruit juice aliquots were stored at −20 °C until analysis, with no further processing. Right before LC-QTOF/MS analysis, the samples were thawed at room temperature, centrifuged, and filtered through regenerated cellulose (RC) syringe filters. To simulate adulteration, pomegranate juice admixtures with apple and red grape juice were constructed at 1%, 2%, 3%, 5%, 10%, and 20% adulteration. Separate adulterated samples were obtained from the Hicaz and Ermioni pomegranate varieties. Pool juice samples of each fruit were used for the adulteration experiments, prepared by mixing equal portions of individual pomegranate, apple, and red grape samples. All samples were analyzed in triplicate, and the average retention times and peak areas were calculated for each compound. 

Besides the pure and adulterated fruit juice samples, a quality control (QC) sample was also prepared and analyzed periodically throughout the batch to evaluate and ensure adequate analytical performance. The QC sample was constructed by mixing same-volume aliquots of all examined pure juices, representing both the sample matrix and metabolite composition of the samples. It was injected at the beginning of the LC-QTOF/MS analysis (six times for conditioning) and also at regular intervals (every 12 injections) to monitor potential instrumental drifts. Three exact mass retention time (EMRT) pairs (*m/z* 191.0516_1.3 min, *m/z* 489.1973_5.3 min, and *m/z* 304.1924_10.0 min) were monitored in terms of peak area and retention time (RT) stability, and in all cases the %relative standard deviations (RSDs) were below 15%. 

### 2.3. LC-QTOF/MS Analysis

The analysis of fruit juices was carried out using an ultrahigh-performance liquid chromatography (UHPLC) system with a HPG-3400 pump (Dionex Ultimate 3000 RSLC, Thermo Fischer Scientific, Dreieich, Germany) coupled to a QTOF mass spectrometer (Maxis Impact, Bruker Daltonics, Bremen, Germany). Chromatographic separation was performed using an Acclaim RSLC C18 column (2.1 × 100 mm, 2.2 μm) from Thermo Fischer Scientific (Dreieich, Germany) preceded by a C18 guard column thermostatted at 30 °C. The mobile phase consisted of water/methanol (90:10 *v/v*, solvent A) and methanol (solvent B), both containing 5 mM of ammonium acetate, and the gradient elution program started with 1% B (flow rate of 0.2 mL min^−1^) for 1 min, which was increased to 39% in 2 min and then to 99.9% (flow rate of 0.4 mL min^−1^) in another 11 min. Here, 99.9% of B was kept constant for 2 min (flow rate of 0.48 mL min^−1^), and then re-equilibration of the column was performed, restoring the initial conditions for 3 min. The injection volume was set up to 5 μL. Ionization was performed using an electrospray ionization interface (ESI), operating in negative mode, with the following operation parameters: A capillary voltage of 3500 V, a nebulizer gas pressure of 2 bar (N_2_), drying gas at 8 L min^−1^, an end-plate offset of 500 V, and a dry temperature of 200 °C.

For each sample, the full scan mass spectra were obtained in a range of 50–1000 *m/z* using Bruker broadband collision-induced dissociation (bbCID) mode. The Bruker bbCID function offers MS and MS/MS spectra within the same injection, with a scan rate of 2 Hz working at two different collision energies (CEs), one low (4 eV) and one high (25 eV). This mode provides high MS sensitivity, enabling the determination of even low-concentration marker compounds that can differentiate fruit juices and reveal juice-to-juice adulteration. However, the acquired MS/MS spectra were noisy and not compound-specific, rendering the identification of compounds rather difficult. For this reason, a second MS analysis was performed in AutoMS (data-dependent) acquisition mode. In AutoMS, the five most abundant ions per MS scan are selected and fragmented, and the applied collision energy is set to predefined values based on the mass and charge state of the ions. This mode provided clear and compound-specific MS/MS spectra, which were used for the structure elucidation of unknown marker compounds. For low-concentration marker compounds that were not within the five most abundant ions per MS scan and where no MS/MS spectra were obtained, a third MS analysis was performed using a preselected inclusion mass list containing the precursor ions of interest (exact masses). The fragmentation of these *m/z* was triggered when their MS spectra intensity exceeded a specific intensity threshold. 

A QTOF external calibration was performed daily using sodium formate in a mixture of water:isopropanol (50:50 *v/v*), and also internal calibration was performed by calibrant injection at the beginning of each run (1st segment, 0.1−0.25 min). A typical resolving power (Full width at half maximum, FWHM) between 36,000 and 40,000 at *m/z* 226.1593, 430.9137, and 702.8636 was provided. The TASQ 1.4 and Data Analysis 4.1 Bruker Daltonics software packages (Bremen, Germany) were used for mass spectra interpretation and data processing.

### 2.4. Screening Strategies 

#### 2.4.1. Target Screening

Among fruit secondary metabolites, phenolic compounds constitute a wide class of biomarkers, and their study has proven to be a powerful tool for assessing fruit juice authentication. Phenolic profiling has provided very promising results concerning the detection of juice-to-juice adulteration, as specific variations in a juice’s phenolic profile can confirm which fruits are present [25,26]. Thus, our study was targeted mainly at the detection and identification of unique phenolic compounds that could serve as markers for the presence of apple and red grape juice in pomegranate juice.

A target database was built that included 37 phenolic compounds from different classes (flavones, flavonols, flavanols, flavanones, and phenolic acids) for which reference standards were commercially available. The database included information on the analytes’ molecular formulas, pseudomolecular ions [M-H]^−^, retention times, and MS/MS fragments (qualifier ions) and is presented in Table S1 in “Electronic Supplementary Materials”. Identification of the target compounds in the samples was performed on the basis of mass accuracy, isotopic fitting, retention time, and MS/MS fragments, and specific criteria thresholds were set. The mass error should not have exceeded 2 mDa for both the precursor ion and the qualifier ions, while mSigma values, measuring the isotopic fitting between the measured and theoretical molecular formulas, should have been below or equal to 50. The retention time tolerance threshold was set at ±0.2 min, the minimum peak area threshold at 800, and the minimum intensity threshold at 200, as reported in a previous study by our group [27]. Quantification of the analytes in pure and adulterated juice samples was performed through an external standard calibration method using standard solution calibration curves. 

The developed LC-QTOF/MS target methodology was validated in order to verify its suitability for identification and quantification purposes. The validation was performed using pomegranate juice samples spiked with different concentrations of the targeted compounds. Linearity was evaluated using standard solutions, prepared as described in Section 2.1, and the intraday precision of the analyses was calculated by analyzing six replicates of spiked pomegranate samples at a concentration level of 5 mg/L. The method limits of detection (MLODs) and method limits of quantification (MLOQs) were defined as the analyte’s concentration at which the signal-to-noise ratio (S/N) was above 3 and 10, respectively, and the matrix effect was evaluated by comparing standard solutions of the analytes prepared in pure solvent and in pomegranate juice samples according to the following equation:

%Matrix Effect = ((Peak area matrix matched standard/Peak area standard in pure solvent) – 1) × 100.(1)

#### 2.4.2. Nontarget Screening 

Initially, LC-HRMS raw data files of all 29 samples analyzed were converted to mzXML files using ProteoWizard open source software (Proteowizard, Palo Alto, CA, USA). These files were transferred to the R environment and processed with an XCMS package using the centWave method for peak picking. The CentWave feature detection algorithm has been successfully used for LC-HRMS data, directly detecting regions of interest (ROIs) in the *m/z* domain [28]. XCMS peak picking parameters such as tolerated mass deviation (“ppm”) and minimum and maximum chromatographic peak width (“min peakwidth, max peakwidth”) were optimized using the IPO package in the R environment [29], and the optimized parameter results were 23.3, 17.5, and 40, respectively. The chromatographic signal-to-noise threshold (“snthresh” parameter) was set at a default value of 3 to filter noisy peaks. A prefilter (intensity filter defined as the threshold for an *m/z* to be considered a peak appearing in *k* consecutive scans at *J* intensity threshold (*k*,*J*)) was adjusted at (31,000) to discard false peaks early in the detected ROIs. A retention time correction was performed using a nonlinear retention time alignment wrapping algorithm through loess, and a final step of filling in the missing peaks was implemented to replace the missing values of nondetected peaks with a small value of the intensity [30]. Finally, the CAMERA and Non-target R packages were used complementarily for the annotation of isotope and adduct peaks [31,32]. 

After peak picking, a differential analysis was performed between authentic pomegranate, apple, and red grape fruit juices, which were processed in pairs. Nonparametric independent (unpaired) two-group tests (one-way analysis of variance (ANOVA) and Welch’s *t*-test) were used to find the mass features (including accurate mass values and retention times) that differentiated pomegranate juice from apple juice and pomegranate juice from red grape juice. An unpaired differential analysis was selected because the two authentic juices were expected to have different chemical profiles (peak area or intensity measurements of detected compounds) and there was no knowledge about the parameters of data distribution between two groups [33]. In general, two-group tests allow for the determination of metabolite features whose levels are significantly different between two sets of samples. Here, fold changes (variations in the maximum intensity of *m/z* values at a given retention time between two groups), *p*-values (to filter in the *m/z* values whose intensity/peak area changes were significant between two groups), and Welch’s *t*-test (to derive the group-regulated data for each *m/z*) were used [34,35]. 

Following the application of a nontarget screening workflow, a large dataset consisting of mass features (including accurate mass values and retention times) that discriminated pomegranate from apple and red grape juice samples was obtained. Mass features that were detected in apple and red grape but not in pomegranate juice or that presented great differences in abundance (more than 5 times higher abundance in apple and grape juice) were selected as *m/z* markers of interest, as they could reveal potential pomegranate juice adulteration. Two suspect databases were compiled, including the mass features of interest for each adulterant, and pomegranate–apple and pomegranate–grape adulterated samples were screened accordingly. The mass features that were determined in the adulterated pomegranate juices presented unique authenticity markers, unmasking pomegranate juice adulteration at different adulteration levels. 

Selected *m/z* markers were tentatively identified according to their mass accuracy (<5 mDa), isotopic fit, MS/MS fragmentation pattern, and retention time. Elemental compositions of precursors and fragment ions were proposed, and probable molecular formulas were suggested using the Bruker Smart Formula Manually tool in Data Analysis 4.1. MS/MS spectra were examined and interpreted using literature data, spectral libraries such as MassBank [36], an online database search (FoodB, METLIN, and CHEBI), and in silico fragmentation tools, mainly Metfrag [37]. 

### 2.5. Chemometric Analysis

A multivariate statistical analysis was performed using unsupervised and supervised pattern chemometric techniques (PCA and PLS-DA) through an in-house program called ChemoTrAMS [38] in the R environment (RStudio, Version 1.1.463, Boston, MA, USA). A PCA was applied to the data obtained from target analysis and was used to locate any existing clustering of fruit juices based on their composition (pomegranate, apple, and grape juices) and their authenticity (pure or adulterated pomegranate juices). A PCA was used as an initial descriptive approach, while PLS-DA, as a supervised method, was applied to construct the supervised classification and prediction models. For this purpose, a dataset was constituted that included the variables (markers) obtained from nontarget screening in both authentic and adulterated pomegranate juice samples. The autoscaling method was used to remove any variation comprised during analysis (such as a loss of instrumental sensitivity) of an original HRMS peaks list. PLS-DA models were built and were able to determine the percentage of adulteration in pomegranate fruit juices and also the adulterant (apple or red grape). The reliability of the classification models was studied in terms of goodness-of-fit (*R*^2^, recognition ability) and goodness-of-prediction (*Q*^2^, prediction ability).

## 3. Results and Discussion

### 3.1. Target Screening

The developed targeted LC-QTOF/MS methodology was used to screen authentic and adulterated pomegranate, apple, and red grape fruit juices. Eighteen compounds were determined: Six phenolic acids (gentistic acid, caffeic acid, cinnamic acid, ferulic acid, *p*-coumaric acid, salicylic acid), eight flavonoids (epicatechin, eriodictyol, myricetin, naringenin, quercetin, taxifolin, catechin, rutin), two phenolic alcohols (tyrosol and hydroxytyrosol), one stilbenoid (resveratrol), and one phenolic aldehyde (syringaldehyde). For all of the determined compounds, the mass accuracies of both precursor ions and qualifier ions were <2 mDa compared to standard solutions, and also the mSigma value (isotopic fit) was <50. Quantification of the compounds was performed using their corresponding standard solution calibration curves, and the concentrations of the phenolic compounds determined were calculated as the average value ± the standard deviation of triplicate analyses for each sample. In every case, the %RSD of the three replicates did not exceed 10% for each individual sample. The target screening results for the authentic fruit juices examined are presented in Table 2. 

**Figure 1:**
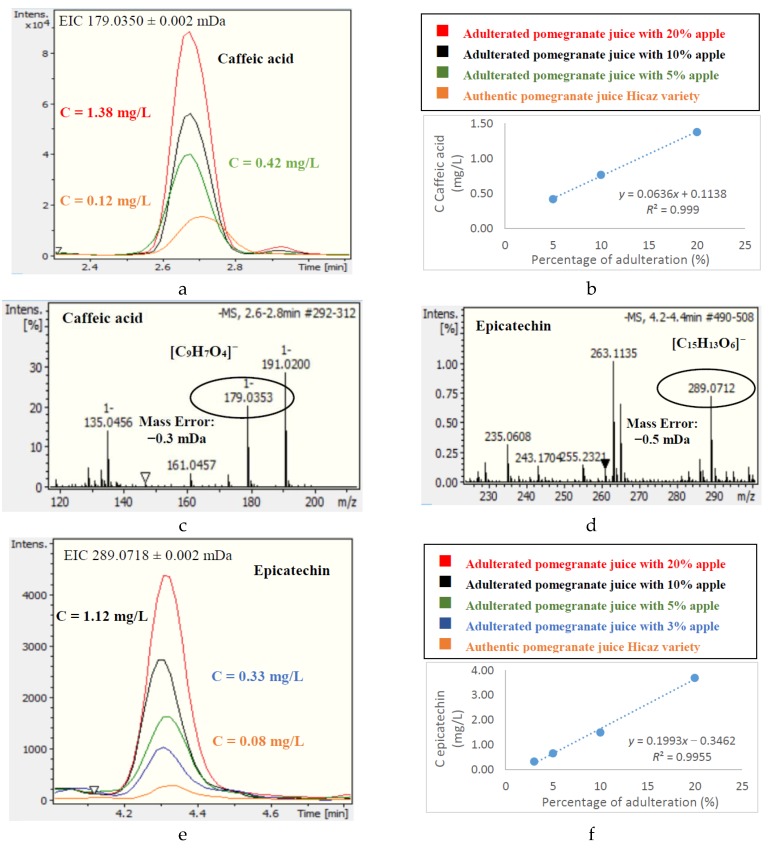
Extracted Ion Chromatograms and Mass Spectrometry (MS) spectra of epicatechin and caffeic acid in authentic and adulterated Hicaz pomegranate juices. (**a**) EIC of caffeic acid in authentic and adulterated pomegranate juice. (**b)** Linear regression of caffeic acid and adulteration percentage. (**c**) MS spectra of caffeic acid. (**d**) MS spectra of epicatechin. (**e**) EIC of epicatechin in authentic and adulterated pomegranate juice. (**f**) Linear regression of epicatechin and adulteration percentage.

The most abundant polyphenolic compounds in apple juices were found to be epicatechin and caffeic acid, with the contents of rutin and catechin being relatively lower. These results were in agreement with previous reported results [39,40,41]. Epicatechin and caffeic acid were detected in significantly lower amounts in Hicaz pomegranate juice, suggesting that they could be used as potential markers to differentiate the two juices and reveal Hicaz pomegranate juice adulteration from apple juice. Indeed, adulteration experiments showed that the presence of epicatechin at a concentration ≥0.25 mg/L (three times higher than in authentic samples) was indicative of pomegranate juice adulteration corresponding to the addition of at least 3% apple juice. The same applied for caffeic acid, for which a concentration ≥0.36 mg/L indicated apple juice addition to pomegranate juice of 5% or more. The concentrations of both epicatechin and caffeic acid were found to have a linear correlation with the percentage of apple juice added to pomegranate juice. These results are presented in Figure 1. However, epicatechin could not be used as a marker to detect apple juice addition in the Ermioni variety of pomegranate juice, as this variety presented a significant amount of epicatechin (2.2 mg/L). Ermioni pomegranate juice debasing with apple juice could be revealed by the presence of caffeic acid at a level as low as 1%, since no caffeic acid was detected in the Ermioni variety of pomegranate juice (Figure S1). 

Pomegranate juice adulteration from red grape juice could be detected based on the concentrations of epicatechin, catechin, hydroxytyrosol, and resveratrol. The presence of epicatechin in Hicaz pomegranate juice could reveal adulteration from red grape juice as low as 2% (at concentrations ≥0.25 mg/L), while catechin was a relatively less indicative marker, as it also exists in pomegranate juice in lower quantities (adulteration ≥5%). However, neither catechin nor epicatechin could be used as adulteration markers in Ermioni pomegranate juices, as they both exist in high amounts in this variety (Figure S2). Hydroxytyrosol proved to be a characteristic marker of pomegranate adulteration from red grape juice, as it could disclose 3% adulteration or higher (concentration (C) ≥ 0.35 mg/L) in both examined pomegranate varieties, which also applied for resveratrol (20% adulteration, C ≥ 0.15 mg/L) (Figure S3). 

The performance of the developed UPLC-QTOF/MS target method was validated to ensure its suitability for identification and quantification purposes. Various analytical parameters were examined, including accuracy (recovery), precision (%RSD), limits of detection and quantification (LODs and LOQs), linearity (calibration curves) and matrix effects, the results for which are presented in Table S2 in the Electronic Supplementary Materials. Intraday precision was assessed in terms of the %RSD, which varied from 0.92% (chrysin) to 7.25% (salicylic acid) and proved the excellent repeatability of the proposed methodology. Calibration curves were constructed in a concentration range from 0.25 to 10 mg/L, displaying excellent linearity with correlation coefficients >0.99 for all analytes. All target compounds showed adequate recovery efficiency (58.8% for gallic acid to 103% for 2’,4’-dihydroxychalcone), and relatively low matrix effects were observed, with 30 out of 37 compounds presenting matrix effects ±40%. The LODs and LOQs were satisfactory, ranging between 0.0095 mg/L (hesperetin) and 0.087 mg/L (catechin) and 0.029 and 0.26 mg/L, respectively. 

### 3.2. Nontarget Screening

The application of the nontarget screening workflow and the differential analysis in pomegranate and apple juice samples produced 1054 *m/z* markers that were detected in apple juice but not in pomegranate juice (or demonstrated a great difference in abundance). A fold value threshold of 10 was applied in order to distinguish the most robust and reliable markers responsible for differentiating the fruit juices, and 214 *m/z* markers were further investigated to evaluate their usefulness as adulteration markers. An in-house suspect list that included these *m/z* markers was built, and all authentic and adulterated pomegranate juice samples were screened using TASQ 1.4 from Bruker. Four out of 214 important mass features already existed in the target list (epicatechin, catechin, caffeic acid, and rutin) and thus were excluded from the nontarget list. From the 214 mass features investigated, 67 could disclose 20% adulteration or more, as they exhibited at least three times higher abundance in adulterated samples compared to authentic ones. Similarly, 48 mass features were indicative of 10% adulteration, 28 of 5%, 14 of 3%, 27 of 2%, and 3 mass features could even reveal 1% adulteration of pomegranate juice from apple juice (Table S3). From the annotation of these 67 mass features, and after excluding adducts, isotopes, and in-source fragments, 42 marker compounds were determined to reveal pomegranate juice adulteration from apple juice. Additionally, five mass features were identified as double-charged compounds (*m/z* 588.1883_3.4 min, *m/z* 446.0816_1.0 min, *m/z* 728.2276_3.4 min, *m/z* 609.1929_3.3 min, and *m/z* 579.1475_1.0 min), as they revealed characteristic isotopic patterns.

For pomegranate–red grape juice samples, 1335 *m/z* markers characteristic of grape juice were obtained. Following the same procedure, 191 significant mass features were included in the second in-house suspect list, which was used to screen all authentic and adulterated pomegranate juice samples. Six out of these 191 mass features already existed in the target list (epicatechin, catechin, hydroxytyrosol, salicylic acid, taxifolin, and resveratrol). After a careful examination of the dataset of adulterated pomegranate juice samples from grape juice, 47 *m/z* markers were found to disclose adulteration at a level of 20% or more; 37 at a level of 10%; 17, 10, and 4 *m/z* markers at levels of 5%, 3%, and 2%, respectively; and, finally, 3 *m/z* markers were able to reveal pomegranate juice adulteration even at a level of 1%. Annotation of these mass features led to the determination of 45 marker compounds, as two mass features (*m/z* 163.0401_1.7 min and *m/z* 203.1076_6.6 min) were found to belong to in-source fragments of other marker compounds. All results are presented in Table S4 in “Electronic Supplementary Materials”.

### 3.3. Tentative Identification of Marker Compounds 

The identification of the characteristic markers that discriminated between adulterated and authentic pomegranate juices represented one of the most difficult and challenging steps of the metabolomics workflow. The use of LC-HRMS-QTOF instrumentation ensured the acquisition of accurate MS and MS/MS spectra, which were essential for the reliable elemental formula estimation of mass features of interest. The probable elemental compositions of marker compounds were computed using “SmartFormula Manually” from Bruker, which is based on accurate mass determination and isotopic patterns. C (*n* ≤ 50), H (*n* ≤ 100), O (*n* ≤ 20), N (*n* ≤ 10), and S (*n* ≤ 5) atoms were considered for the molecular formula calculations. The proposed formulas were sorted according to the SmartFormula Manually Score (the most probable proposed formula scored 100%). Subsequently, a stepwise search of the biomarkers’ proposed molecular formulas (in a descending order) was performed in several online databases, such as MassBank (http://www.massbank.jp/?lang=en), METLIN (https://metlin.scripps.edu/landing_page.php?pgcontent=mainPage), ChEBI (https://www.ebi.ac.uk/chebi/), and FoodB (http://foodb.ca/), with the latter more focused on natural product constituents. Only candidates that could possibly be present in fruit juices were further examined, and the experimental MS/MS mass spectra were compared to those provided in the databases and/or literature. In silico fragmentation with Metfrag [37] was also performed to elucidate the chemical structure of potential biomarkers. 

**Figure 2:**
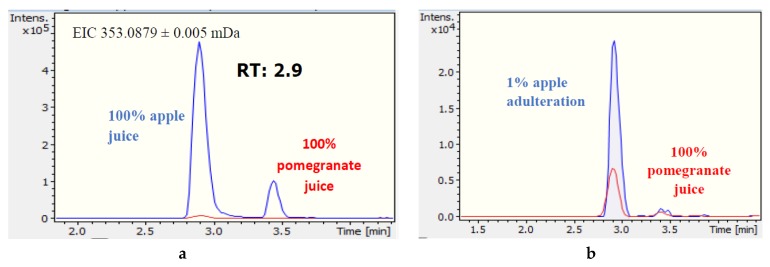

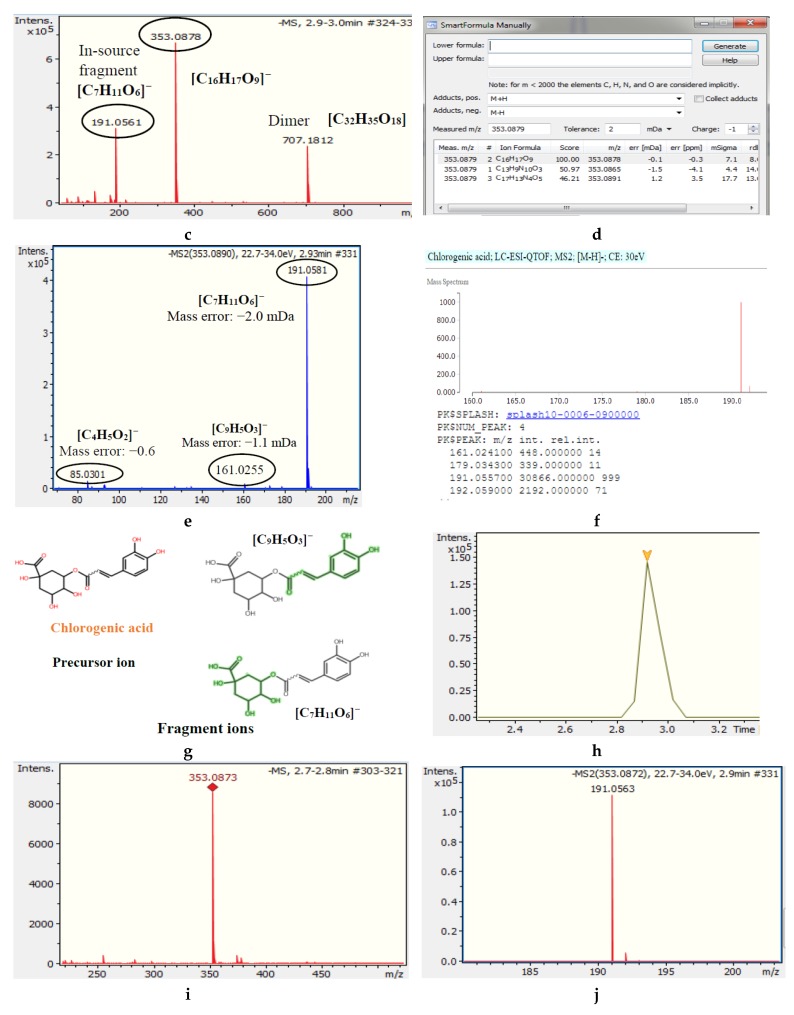
Identification data for the mass feature *m/z* 353.0878_2.9 min (chlorogenic acid). (**a**) EIC of *m/z* 353.0879 in apple–pomegranate juice. (**b**) EIC of *m/z* 353.0879 in authentic and adulterated pomegranate juice. (**c**) MS spectra of mass feature *m/z* 353.0879_2.9 min. (**d**) Probable elemental composition of mass feature *m/z* 353.0879_2.9 min. (**e**) MS/MS spectra of mass feature *m/z* 353.0879_2.9 min. (**f**) MS/MS spectra of chlorogenic acid (MassBank record FIO00625). (**g**) Precursor and fragment ions of chlorogenic acid. (**h**) EIC of *m/z* 353.0879 in the chlorogenic acid reference standard. (**i**) MS spectra of chlorogenic acid. (**j**) MS/MS spectra of chlorogenic acid.

For pomegranate juice adulteration with apple juice, 22 out of 42 marker compounds were tentatively identified. Three EMRTs were found to reveal adulteration down to 1%: *m/z* 353.0879_2.9 min, *m/z* 191.0564_2.9 min, and *m/z* 193.0509_6.1 min. However, these mass features corresponded to only two different marker compounds, as the *m/z* 191.0564_2.9 min ion was proven to be an in-source fragment of *m/z* 353.0879_2.9 min (Figure 2a–c). For the mass feature *m/z* 353.0877_2.9 min, the most probable molecular formula was suggested to be C_16_H_18_O_9_, with a mass error of 0.1 mDa and an mSigma value of 7.1 (Figure 2d). This molecular formula corresponded to 16 potential candidates in the FoodB and Metlin databases, from which only chlorogenic acid and its isomers have been reported to exist in fruits. The experimental MS/MS spectra obtained (Figure 2e) were compared to the MS/MS spectra of chlorogenic acid that are reported in MassBank (Figure 2f), and two common fragments were revealed (Figure 2g). A reference standard was then obtained for chlorogenic acid, and its presence in the samples was confirmed (Figure 2g–i). Following the same workflow, the mass feature with *m/z* 193.0509_6.1 min was tentatively identified as vanillin acetate (Figure S4 in “Electronic Supplementary Materials”). The probable elemental compositions and tentative identification of all marker compounds that revealed pomegranate juice adulteration from apple juice are presented in Table 3. Identification data for selected marker compounds are presented in “Electronic Supplementary Materials”, Figures S4–S12. 

Phloridzin fragments with *m/z* 273.0771_5.9 min and phloretin (*m/z* 273.0766_7.5) were confirmed with reference standards. Although phloridzin is a unique apple juice marker, as is also reported in the literature [26,39], the adulteration of pomegranate juice from apple juice cannot be revealed by monitoring its precursor ion (*m/z* 435.0308). The reason is that an isobaric compound of phloridzin, tentatively identified as phenethyl 6-galloylglucoside, exists in pomegranate juice and is eluted at the same RT as phloridzin (Figure S5e). However, these two compounds can be easily distinguished by their different fragmentations (MS/MS spectra), as is shown in Figure S5d,g. Consequently, phloridzin’s in-source fragment with *m/z* 273.0766 could be accurately used as an adulteration marker, revealing the presence of apple juice in pomegranate juice at a level down to 3% (Figure S5). Moreover, two of the most important markers, with *m/z* 373.0942, RT 3.9, and 3.3 min, were tentatively identified as *p*-coumaroylquinic acid isomers, detecting pomegranate juice adulteration of 2% and 5%, respectively. Both 4-*O*-*p*-coumarylquinic acid and 3-*O*-*p*-coumarylquinic acid have been reported to exist in apple juices, showing characteristic fragmentation patterns [26]. In the absence of reference standards to provide the exact RT and MS/MS spectra, we were unable to distinguish between positional isomers (Figure S6).

**Figure 3:**
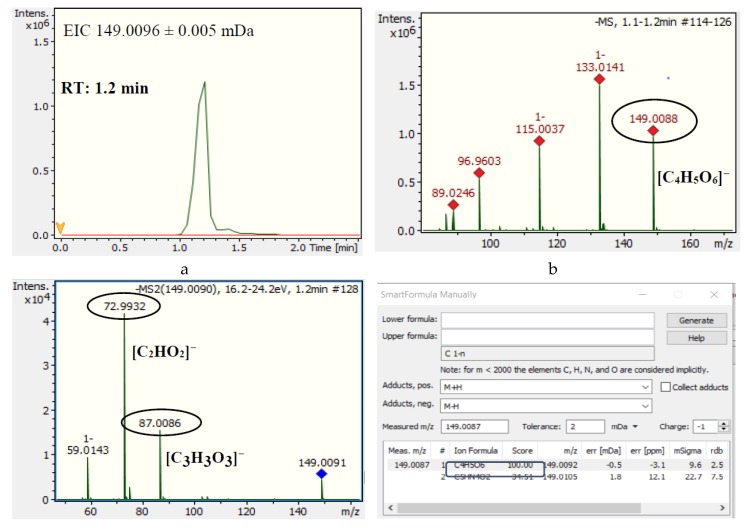

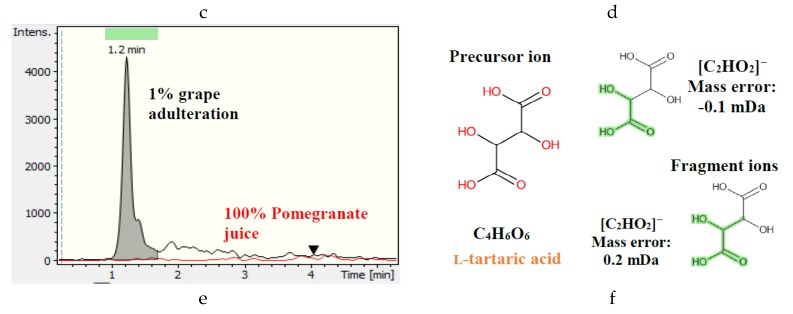
Identification data for the mass feature *m/z* 149.0096_1.2 min (l-tartaric acid). (**a**) EIC of *m/z* 149.0096 in pomegranate–grape juice. (**b**) MS spectra of mass feature *m/z* 149.0096_1.2 min. (**c**) MS/MS spectra of mass feature *m/z* 149.0096_1.2 min. (**d**) Probable elemental composition of mass feature *m/z* 149.0096_1.2 min. (**e**) EIC of *m/z* 149.0096 in authentic and adulterated pomegranate juice samples. (**f**) Structures of precursor and fragment ions of l-tartaric acid.

For pomegranate juice adulteration from grape juice, 18 out of 45 marker compounds were tentatively identified (Table 4). Three mass features were found to reveal adulteration down to 1%: *m/z* 369.0278_2.2 min, *m/z* 149.0096_1.2 min, and *m/z* 287.1502_4.0 min. The mass feature with *m/z* 149.0096_1.2 min was identified as tartaric acid, a well-known constituent of grape juice [2]. As presented in Figure 3, the molecular formula calculated by SmartFormula Manually for this mass feature was C_4_H_7_O_6_, with a mass error of −0.5 mDa. Two characteristic fragment ions of tartaric acid were detected in the MS/MS spectra, C_2_HO_2_^−^ with *m/z* 72.9932 and C_3_H_3_O_3_^−^ with *m/z* 87.0086, recording a score of 1.0 in MetFrag. Following the same workflow, 17 more compounds were identified, among them malvidin glucoside, resveratrol 3-glucoside, cis-coutaric acid, procyanidin B, quercetin 3-glucuronide, protocatechuic acid 4-glucoside, and peonidin 3-glucoside, as presented in Table 4 and Figures S13–S18 of the Electronic Supplementary Materials. Particularly for malvidin-3-*O*-glucoside, two characteristic ions of anthocyanin fragmentation in negative ionization were found in the MS spectra, [M-2H]^−^ with *m/z* 491.1191 and [M-2H + H_2_O]^−^, according to a previous study of Sun et al. [42]. 

### 3.4. Chemometric Analysis

**Figure 4:**
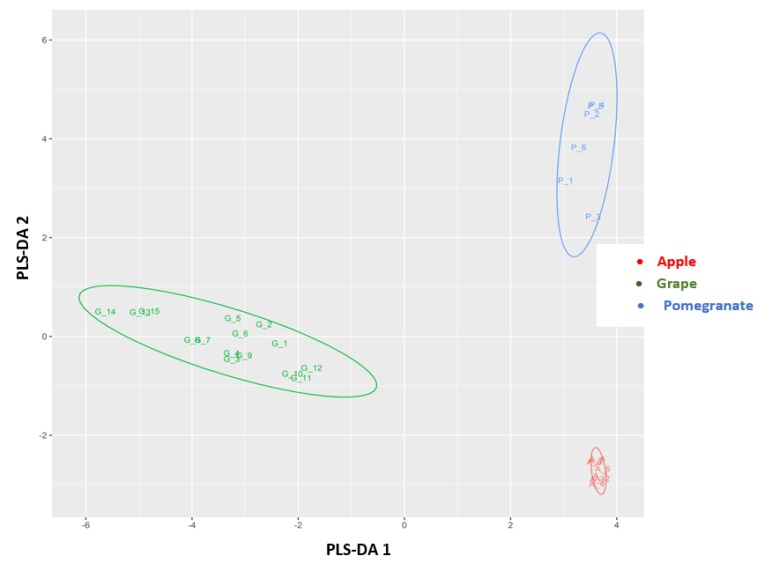
Principal component analysis (PCA) score plot showing the classification of authentic pomegranate, apple, and red grape juice samples.

Initially, the results of target screening methodology were used to differentiate authentic pomegranate, apple, and red grape juice samples. A PCA was performed on the 18 × 29 dataset (18 phenolic compounds were identified and quantified in 29 pure fruit juice samples). The PCA score plot generated for pure pomegranate, apple, and grape juice samples showed a distinctive separation between the three groups, with the first three principal components (PC1, PC2, and PC3) explaining the majority of the variation (58.8%, 25.5%, and 8.8%, respectively) (Figure 4). Subsequently, adulterated pomegranate samples with both apple and red grape juice were included with the existing PCA using the same dataset. However, the explained variance in PCs decreased, and there was no clear separation between adulterated and pure juice samples. 

PLS-DA was then applied to obtain classification models that could distinguish authentic pomegranate juices from adulterated ones in a supervised manner. At first, different models were built to differentiate pure pomegranate juices from those adulterated with apple and red grape juice. For the detection of pomegranate juices adulterated with apple juice, an autoscaled 28 × 51 dataset was used to build the model, in which rows represented the juice samples analyzed (28 objects) and columns the peak areas of the individual marker compounds, which were determined through both target and nontarget LC-QTOF/MS methodologies (51 variables). From the 28 authentic and adulterated juices, 18 juices were selected as the training set and 10 as the test set, randomly. The PLS-DA model correctly classified all authentic and adulterated pomegranate juice samples to a level of adulteration down to 1%. From the loading data, the most significant variables (using variable importance in projection (VIP) [27]) in the PLS-DA model were shown to be vanillin acetate and chlorogenic acid. Subsequently, a second model was built to detect pomegranate juice adulteration from red grape juice, again to a level of adulteration down to 1%. An autoscaled 28 × 50 dataset was used to build the second model (28 juice samples analyzed, 50 markers of red grape adulteration). Nineteen juices were selected in the training set and 9 in the test set, randomly. Again, the PLS-DA model successfully classified all authentic and adulterated pomegranate juice samples, even at the lowest level of adulteration (1%). From the loading data, the VIP variables that were indicative of adulteration from grape juice were hydroxytyrosol and an unknown mass feature with *m/z* 287.1502_4.0 min. 

**Figure 5:**
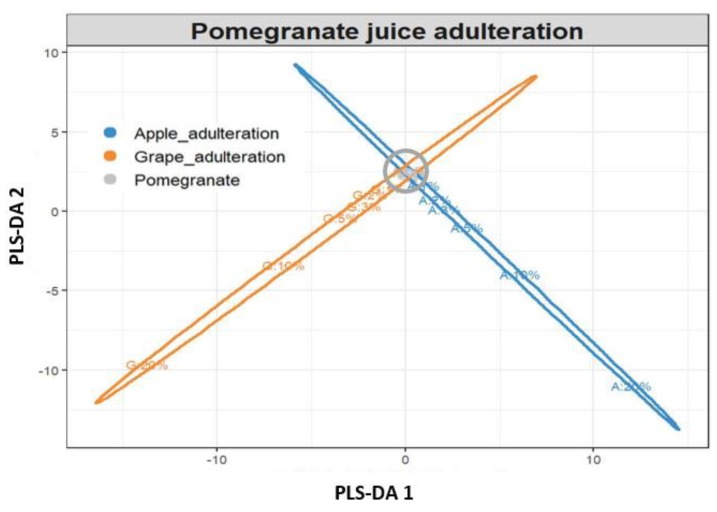
Partial least squares discriminant analysis (PLS-DA) plot showing the classification of authentic and adulterated pomegranate juice samples.

Finally, a third PLS-DA model was built to separate authentic pomegranate juice samples from adulterated ones containing either apple and/or red grape juice as the major adulterant. An autoscaled 52 × 39 dataset was used (42 samples, 39 variables) that included markers of adulteration with both fruit juices to a level down to 5% (Table 3 and Table 4). The training set included 8 pure pomegranate juices (Hicaz cultivar) and also 30 juices adulterated with red grape and apple juice in a range of 20% to 1%. Six replicates of 1% juice adulteration (both red grape and apple) were included in the training set to increase the accuracy of the models at low adulteration levels. In order to evaluate the predictability of the model, the test set included 14 pure and adulterated pomegranate samples belonging to the Hicaz variety, but also authentic and adulterated freshly squeezed pomegranate juices from the Ermioni variety, prepared as described in Section 2.2. The prediction accuracy of the PLS-DA model was found to be more than adequate, as it successfully classified the authentic and adulterated pomegranate juices, even when they belonged to different varieties (Ermioni and Hicaz) (Figure 5). More specifically, the model successfully predicted all the adulteration ratios from red grape juice (down to 1%), while in the case of pomegranate juices adulterated with apple juice, it successfully predicted adulteration down to 2%, misclassifying only the 1% adulterated samples (they were indicated as pure pomegranate juice). The model cross-validation parameters were found to be very robust, with a goodness-of-fit (*R*^2^) and goodness-of-prediction (*Q*^2^) of 0.97 and 0.93, respectively, taking into consideration the first four PLS components. No outliers were observed according to Hotelling’s *T*2 using a control limit of 95%. The results obtained through the PLS-DA models distinctly showed that the markers of adulteration that were detected through this study could be accurately used to achieve successful differentiation of authentic and adulterated pomegranate juices of different varieties. 

## 4. Conclusions

A novel approach was developed for the evaluation of pomegranate juice authenticity based on targeted and untargeted metabolomics coupled with advanced chemometric techniques. The combination of metabolomic profiling, metabolomic fingerprinting, and chemometrics provided a powerful approach for detecting pomegranate juice adulteration from apple and red grape juice at very low adulteration levels (down to 1%). The developed methodology is simple, sensitive, reliable, and robust and could be used not only for research purposes but also as an effective tool for the routine monitoring of pomegranate juice adulteration. To the best of our knowledge, this is the first study in the literature reporting more than 80 potential *m/z* markers that indicated the fraudulent addition of apple and/or grape juice in pure pomegranate juices in different portions. Several of these markers were identified, including phenolic acids, flavonoids, anthocyanins, and other minor metabolites. Processing of the mass spectrometric datasets of the m/z markers in authentic and artificially adulterated pomegranate samples by PCA and PLS-DA led to the construction of reliable and accurate classification and prediction models that could successfully discriminate between authentic and adulterated samples.

## Figures and Tables

**Table 1:** Fruit juice samples.

Juice	Variety	Origin	°Brix	Sample Code
Apple	Starkin, Granny Smith	Greece (Pella, Imathia, Kastoria, Larissa)	11.2 ± 0.5	A1–A8
Red grape	Sangiovese, Montepulciano, Lambrusco, Schiava, Shiraz, Ciliegiolo, Merlot	Italy (Puglia)	15.9 ± 0.5	G1–G15
Pomegranate	Hicaz	Turkey	15 ± 0.5	P1–P5
	Ermioni	Greece (Argolida)	15.3	P6

**Table 2:** Concentrations of phenolic compounds in pure pomegranate, apple, and red grape juices. LOQ: Limit of quantification.

Compound	Pomegranate Juice, Hicaz Variety (*n* = 5)	Pomegranate Juice, Ermioni Variety (*n* = 1)	Apple Juice (*n* = 8)	Red Grape Juice (*n* = 15)
	Concentration Range (mg/L)	Concentration (mg/L)	Concentration Range (mg/L)	Concentration Range (mg/L)
Caffeic acid	0.045–0.12	<LOQ	2.9–5.3	0.58–1.4
Catechin	0.71–1.1	4.1	0.94–1.1	12.2–46.3
Cinnamic acid	0.36–0.55	<LOQ	<LOQ	<LOQ
Epicatechin	0.039–0.083	2.2	4.0–8.2	4.4–14
Eriodictyol	0.12–0.18	<LOQ	0.35–0.42	0.10–0.24
Ferulic acid	0.43–0.78	<LOQ	0.19	0.27–0.90
Gentistic acid	1.0–1.6	2.9	0.49–0.61	3.0–5.3
Hydroxytyrosol	<LOQ	<LOQ	<LOQ	2.3–4.4
Myricetin	0.32–0.45	0.24	<LOQ	0.20–0.60
Naringenin	0.20–0.32	0.28	<LOQ	0.16–0.36
*p*-coumaric acid	0.25–0.50	<LOQ	0.30–0.62	0.55–1.3
Quercetin	0.12–0.20	<LOQ	0.031–0.09	0.15–0.43
Resveratrol	<LOQ	<LOQ	<LOQ	0.17–1.09
Rutin	0.29–0.53	LOQ	1.1–2.5	<LOQ
Salicylic acid	<LOQ	0.16	<LOQ	0.56–2.4
Syringaldeyde	0.32–0.44	0.59	<LOQ	<LOQ
Taxifolin	0.032–0.060	0.054	0.040–0.060	0.32–0.84
Tyrosol	0.10–0.18	<LOQ	0.21–0.44	0.45–0.93

**Table 3:** Tentative identification of characteristic marker compounds indicating pomegranate juice adulteration from apple juice.

# Marker	*m/z* (Precursor Ion)	Retention Time (min)	Ion	*m/z* (Fragment Ions)	Probable Elemental Composition	Mass Error (mDa)	Tentative Identification	Indicative Level of Adulteration
1	353.0879	2.9	[M-H]^−^	191.0564; 192.0611; 93.0345	C_16_H_18_O_9_	0.1	Chlorogenic acid	1%
2	193.0509	6.1	[M-H]^−^	133.0289; 178.9993	C_10_H_10_O_4_	0.3	Vanillin acetate	1%
3	353.0880	3.4	[M-H]^−^	191.0565	C_16_H_18_O_9_	0.2	Chlorogenic acid isomer	2%
4	183.0664	3.7	[M-H]^−^	71.0141; 138.0560	C_9_H_12_O_4_	0.1	Unknown compound	2%
5	337.0942	3.9	[M-H]^−^	173.0464; 163.0407; 119.0505	C_16_H_18_O_8_	1.4	*p*-coumaroylquinic acid	2%
6	191.0551	1.3	[M-H]^−^	85.0302; 72.9937; 127.0411	C_7_H_12_O_6_	1.00	Quinic acid	3%
7	273.0771	5.9	[M-H]^−^	167.0357; 123.0467; 125.0249	C_15_H_14_O_5_	−0.3	Phloridzin (in-source fragment)	3%
8	307.1762	6.3	[M-H]^−^	161.0464; 71.0132	C_14_H_28_O_7_	0.1	(R)-1-*O*-b-d-glucopyranosyl-1,3-octanediol	3%
9	351.1309	3.9	[M-H]^−^	101.0613; 249.0630; 291.1094	C_14_H_24_O_10_	−1.2	2-*O*-acetyl-α-d-abequopyranosyl-(1→3)-α-d-mannopyranose	3%
10	161.0819	2.1	[M-H] ^-^	130.088; 109.0297; 153.0215	C_7_H_14_O_4_	−0.6	Unknown compound	3%
11	517.2284	4.5	[M-H] ^-^	385.1865; 205.1236; 149.0457; 293.0879	C_24_H_38_O_12_	0.7	Vomifoliol 9-[xylosyl-(1->6)-glucoside]	5%
12	165.0776	1.5	[M-H]^−^	89.0247; 119.0359; 149.047	C_6_H_14_O_5_	−0.7	l-rhamnitol	5%
13	195.0882	1.5	[M-H]^−^	71.0142; 59.0141; 73.0298	C_7_H_16_O_6_	−0.4	Unknown compound	5%
14	289.0830	2.8	[M-H]^−^	245.0934; 203.0828; 116.0499	C_14_H_14_N_2_O_5_	0.0	N2-malonyl-d-tryptophan	5%
15	337.0942	3.3	[M-H]^−^	191.0576; 163.042; 119.0512	C_16_H_18_O_8_	−1.3	*p*-coumaroylquinic acid isomer	5%
16	405.1778	3.5	[M-H]^−^	225.1153; 181.1243; 71.0149	C_18_H_30_O_10_	−1.3	Unknown compound	5%
17	393.1768	5	[M-H]^−^	125.0255; 161.0444; 249.1363	C_17_H_30_O_10_	−0.2	Unknown compound	5%
18	425.167	3.9	[M-H]^−^	235.1203; 143.0386; 287.0537	C_17_H_30_O_12_	−0.6	Unknown compound	5%
19	498.1270	5.9	[M-H]^−^	273.078; 167.0364; 307.1781	C_36_H_19_O_3_	−0.9	Phloridzin-related compound	5%
20	351.1299	3.6	[M-H]^−^	191.0595; 71.0143; 101.0622	C_14_H_24_O_10_	−1.2	Unknown compound	10%
21	451.1243	5.3	[M-H]^−^	289.0718; 167.0355; 125.0249	C_21_H_24_O_11_	0.3	3-Hydroxyphloretin 2ʹ-*O*-glucoside	10%
22	517.3162	7.4	[M-H]^−^	285.0398; 383.2585	C_30_H_46_O_7_	−0.8	Corosin	10%
23	429.1769	3.7	[M-H]^−^	205.1250; 249.1141; 161.1353	C_20_H_30_O_10_	−0.4	Unknown compound	10%
24	469.2284	5.8	[M-H]^−^	273.0772; 300.029; 433.0792;	C_20_H_38_O_12_	−0.5	In-source fragment of compound with *m/z* 529.2496	10%
25	456.151	4.1	[M-H]^−^	145.0311; 133.0144;	C_20_H_27_NO_11_	−0.1	Unknown compound	10%
26	497.2234	5.1	[M-CH_3_COOH]^−^	305.1618; 131.0358;179.0577	C_21_H_38_O_13_	−0.6	Ebracteatoside D	10%
27	567.1720	5.5	[M-H]^−^	273.0774; 167.0359; 125.0252	C_26_H_32_O_14_	−0.1	Phloretin 2ʹ-xyloglucoside	10%
28	510.0888	6.0	[M-H]^−^	300.0285; 301.0358; 447.0952	C_21_H_21_NO_14_	0.1	Unknown compound	10%
29	439.2180	6.0	[M-H]^−^	307.1703	C_19_H_36_O_11_	0.5	Unknown compound	10%
30	413.1306	1.5	[M-H]^−^	235.0521	C_15_H_26_O_13_	−0.6	Unknown compound	20%
31	467.1191	2.0	[M-H]^−^	305.0717	C_21_H_24_O_12_	−0.4	Unknown compound	20%
32	583.1663	4.9	[M-H]^−^	167.0354; 289.0717; 125.0240	C_26_H_32_O_15_	0.5	3-hydroxyphloretin 2’’-*O*-xylosylglucoside	20%
33	597.1814	5.2	[M-H]^−^	273.0796; 167.0361; 179.0388	C_27_H_34_O_15_	1.1	Phloridzinyl glucoside	20%
34	485.2236	6.0	[M-H]^−^	59.0142; 71.0145	C_20_H_38_O_13_	0.4	Unknown compound	20%
35	273.0766	7.5	[M-H]^−^	167.0357; 123.0467; 145.0355	C_15_H_14_O_5_	0.2	Phloretin	20%
36	475.1313	1.4	[M-H]^−^	133.0141; 115.0034; 179.0564	C_32_H_56_O_32_	−0.8	Unknown compound	20%
37	207.0652	7.0	[M-H]^−^	161.0272; 133.0292; 179.0381	C_11_H_12_O_4_	1.0	Unknown compound	20%
38	337.1147	3.1	[M-H]^−^	249.0616; 87.0454; 175.0076	C_13_H_22_O_10_	−0.7	2,2-bis[[3-hydroxy-2-(hydroxymethyl)-2-methyl-propanoyl]oxy]propanoic acid	20%
39	425.2025	5.7	[M-H]^−^	326.0673	C_18_H_34_O_11_	0.3	Unknown compound	20%
40	463.0883	5.5	[M-H]^−^	300.0288; 271.0268; 151.0016	C_21_H_20_O_12_	−0.1	Quercetin 3-galactoside	20%
41	501.3215	10.6	[M-H]^−^	483.3176; 409.3087; 483.3118	C_30_H_46_O_6_	−0.6	Esculentic acid	20%
42	580.2237	4.5	[M-H]^−^	149.0467; 205.1241	C_21_H_42_O_18_	−1.7	Unknown compound	20%

**Table 4:** Tentative identification of characteristic marker compounds indicating pomegranate juice adulteration from red grape juice.

# Marker	*m/z* (Precursor Ion)	Retention Time (min)	Ion	*m/z* (Fragment Ions)	Probable Elemental Composition	Mass Error (mDa)	Tentative Identification	Indicative Level of Adulteration
1	369.0278	2.2	[M-H]^−^	125.0240; 161.0240; 80.9650	C_18_H_10_O_9_	−2.6	Unknown compound	1%
2	149.0096	1.2	[M-H]^−^	72.9932; 87.0086; 59.0143	C_4_H_6_O_6_	0.4	l-Tartaric acid	1%
3	287.1502	4.0	[M-H]^−^	227.1283; 123.045; 203.0718	C_14_H_24_O_6_	0.6	Unknown compound	1%
4	491.1191	4.7	[M-H]^−^	328.0586; 329.0645; 313.0343; 330.0682	C_23_H_25_O_12_	0.4	Malvidin-3-*O*-glucoside	2%
5	261.0405	4.9	[M-H]^−^	125.0243; 61.9890; 197.0447; 204.1144	C_13_H_10_O_6_	−0.2	Maclurin	3%
6	389.1242	4.8	[M-H]^−^	227.0716; 185.0597; 143.0515	C_20_H_22_O_8_	−0.9	Resveratrol 3-glucoside (cis-piceid )	3%
7	295.0464	1.7	[M-H]^−^	163.0396; 119.0499; 87.0088	C_13_H_12_O_8_	−0.4	cis-Coutaric acid	3%
8	283.0396	2.7	[M-H]^−^	142.0659; 222.0223; 241.004	C_19_H_8_O_3_	0.5	Unknown compound	3%
9	261.1344	3.0	[M-H]^−^	73.0299; 187.0968; 201.1122	C_12_H_22_O_6_	−0.1	Phaseolic acid	3%
10	311.0808	2.3	[M-H]^−^	185.1174; 80.9647; 130.086; 229.1068	C_15_H_12_N_4_O_4_	−2.2	Unknown compound	3%
11	369.0278	3.0	[M-H]^−^	125.0244;161.0242; 287.0564	C_18_H_10_O_9_	−2.6	Unknown compound	3%
12	577.1346	3.3	[M-H]^−^	289.0712; 125.0245; 407.0739; 161.0239; 245.0812	C_30_H_26_O_12_	0.6	Procyanidin B isomer	3%
13	427.0340	4.1	[M-H]^−^	347.0732; 165.0189; 261.0757	C_13_H_16_O_16_	2.6	Unknown compound	5%
14	477.0671	5.0	[M-H]^−^	301.0341; 151.0031; 178.9983; 316.0197	C_21_H_18_O_13_	1.0	Quercetin 3-glucuronide	5%
15	509.1298	3.3	[M+H20-H]^−^	149.0238; 329.0653; 193.0139; 165.0191; 347.0758	C_23_H_26_O_13_	0.3	Quercetin 3,3’-dimethyl ether 4’-glucoside	5%
16	167.0348	5.5	[M-H]^−^	123.0443; 81.0343	C_8_H_8_O_4_	0.2	Unknown compound	5%
17	295.0858	3.8	[M-H]^−^	169.1227; 80.9653; 213.1121; 170.1277	C_11_H_20_O_7_S	−0.1	Unknown compound	5%
18	315.0725	2.0	[M-H]^−^	152.0113; 255.2329; 217.0038	C_13_H_16_O_9_	−0.3	Protocatechuic acid 4-glucoside	10%
19	121.0293	2.6	[M-H]^−^	59.0144;66.0351	C_7_H_6_O_2_	0.7	Benzoic acid	10%
20	397.0235	4.1	[M-H]^−^	317.0653; 165.0190; 193.0141	C_12_H_14_O_15_	2.5	Unknown compound	10%
21	295.0857	4.5	[M-H]^−^	169.1229; 80.9649; 213.1120	C_15_H_12_N_4_O_3_	−2.1	Unknown compound	10%
22	461.1088	4.6	[M-H]^−^	299.0550; 298.0482; 283.0248; 284.0319	C_22_H_22_O_11_	0.1	Peonidin 3-glucoside	10%
23	231.1027	5.4	[M-H]^−^	169.1018; 213.0925	C_14_H_16_O_3_	0.00	Unknown compound	10%
24	219.1027	6.6	[M-H]^−^	149.0956; 59.0149	C_13_H_16_O_3_	0.00	Unknown compound	10%
25	637.1555	6.9	[M-H]^−^	329.0658; 328.0569; 313.0351	C_32_H_30_O_14_	0.8	Malvidin 3-(6-p-coumarylglucoside)	10%
26	423.0720	7.8	[M-H]^−^	393.0249; 408.0440; 365.0305	C_22_H_16_O_9_	0.1	Unknown compound	10%
27	591.1022	2.0	[M-H]^−^	329.0654; 347.076; 411.0374	C_26_H_24_O_16_	−3.0	Unknown compound	10%
28	446.0759	2.4	[M-H]^−^	222.0219; 142.0658; 266.0106	C_20_H_17_NO_11_	−3.0	Unknown compound	10%
29	369.0288	3.2	[M-H]^−^	125.0239;161.0233; 165.0192	C_18_H_10_O_9_	−2.6	Unknown compound	10%
30	190.0541	2.8	[M-H]^−^	142.0463; 87.0080; 174.9927	C_10_H_9_NO_3_	−3.1	Unknown compound	10%
31	577.1346	3.8	[M-H]^−^	289.0705; 125.0241; 407.0739; 245.0812	C_30_H_26_O_12_	0.6	Procyanidin Β isomer	10%
32	305.0303	3.8	[M-H]^−^	151.0041; 169.0143; 65.0031	C_14_H_10_O_8_	0.1	Unknown compound	10%
33	161.0818	2.1	[M-H]^−^	71.0516; 99.0847	C_7_H_14_O_4_	0.2	Unknown compound	10%
34	209.0304	1.1	[M-H]^−^	59.0139; 71.0142; 85.0347	C_6_H_10_O_8_	0.2	Unknown compound	10%
35	293.1030	6.6	[M-H]^−^	203.1071; 175.1126; 129.0550	C_15_H_18_O_6_	0.1	Unknown compound	10%
36	429.2132	3.4	[M-H]^−^	329.0667; 347.0770	C_21_H_34_O_9_	−0.2	Unknown compound	10%
37	243.1239	3.6	[M-H]^−^	61.9887; 73.0299; 125.0952;	C_12_H_20_O_5_	−0.1	Unknown compound	20%
38	449.1087	5.3	[M-H]^−^	151.0038; 285.0394; 178.9988	C_21_H_22_O_11_	0.2	Taxifolin 3-rhamnoside	20%
39	131.0712	3.1	[M-H]^−^	71.0140; 85.0654	C_6_H_12_O_3_	0.2	Unknown compound	20%
40	330.2037	3.3	[M-H]^−^	129.1035	C_15_H_30_N_3_O_5_	−0.3	Unknown compound	20%
41	366.1198	3.5	[M-H]^−^	125.0976; 142.0668; 187.0979	C_17_H_21_NO_8_	−0.4	Unknown compound	20%
42	107.0502	3.7	[M-H]^−^	67.9611	C_7_H_8_O	0.00	Benzyl alcohol	20%
43	187.0974	3.3	[M-H]^−^	125.0955; 57.0350; 123.0810	C_9_H_16_O_4_	0.1	Azelaic acid	20%
44	373.1143	3.2	[M-H]^−^	193.0504; 178.0269; 343.1009	C_16_H_22_O_10_	−0.3	Geniposidic acid	20%
45	413.2403	5.3	[M-H]^−^	169.0962	C_18_H_38_O_10_	−1.1	Unknown compound	20%

## Supplementary Materials

The following are available online at https://www.mdpi.com/2304-8158/8/6/212/s1, Table S1: Target list of phenolic compounds, Table S2: Validation Data of target screening methodology, Table S3: Mass features revealing pomegranate adulteration with apple in different adulteration levels, Table S4: Mass features revealing pomegranate adulteration with grape in different adulteration levels, Figure S1: EICs and MS spectra of caffeic acid in authentic and adulterated Ermioni pomegranate juices, Figure S2: EICs and MS spectra of catechin and epicatechin in authentic and adulterated pomegranate juices, Figure S3: EICs and MS spectra of hydroxytyrosol and resveratrol in authentic and adulterated pomegranate juices, Figure S4: Identification data for the mass feature *m/z* 193.0509_6.1 min (vanillin acetate), Figure S5: Identification data for the mass feature *m/z* 273.0769_5.9 min (phloridzin in-source fragment), Figure S6: Identification data for the mass features *m/z* 337.0943_3.9 min and *m/z* 337.0943_3.3 min (*p*-coumaroylquinic acid isomers), Figure S7: Identification data for the mass feature *m/z* 191.0551_1.3 min (quinic acid), Figure S8: Identification data for the mass feature *m/z* 307.1762_6.3 min ((R)-1-*O*-b-d-glucopyranosyl-1,3-octanediol, Figure S9: Identification data for the mass feature *m/z* 351.1309_3.9 min (2-*O*-acetyl-alpha-d-abequopyranosyl-(1->3)-alpha-d-mannopyranose), Figure S10: Identification data for the mass feature *m/z* 517.2284 _4.5 min (Vomifoliol 9-[xylosyl-(1->6)-glucoside]), Figure S11: Identification data for the mass feature *m/z* 289.0830 _2.8 min (N2-malonyl-d-tryptophan); Figure S12: Identification data for the mass feature *m/z* 273.0766 _7.5 min (phloretin); Figure S13: Identification data for the mass feature *m/z* 491.1191_4.7 min (malvidin-3-*O*-glucoside), Figure S14: Identification data for the mass feature *m/z* 261.0403 _4.9 min (maclurin), Figure S15: Identification data for the mass feature *m/z* 389.1242 _4.8 min (resveratrol 3-glucoside), Figure S16: Identification data for the mass feature *m/z* 295.0464_1.7 min (cis-coutaric acid); Figure S17: Identification data for the mass feature *m/z* 261.1344_3.0 min (phaseolic acid); Figure S18: Identification data for the mass feature *m/z* 261.1344_3.0 min (procyanidin B).
