# Supplementary material for: Targeted and Untargeted Metabolomics as an Enhanced Tool for the Detection of Pomegranate Juice Adulteration
Source: Foods. 2019 Jun 14;8(6):212. doi: 10.3390/foods8060212 (PMC6617410; doi:10.3390/foods8060212)
Supplement: Supplementary file 1 [file foods-08-00212-s001.pdf]

ELECTRONIC SUPPLEMENTARY MATERIAL

Targeted and Untargeted Metabolomics as an Enhanced Tool for the Detection of Pomegranate Juice Adulteration

Marilena E. Dasenaki*, **Sofia K. Drakopoulou, Reza Aalizadeh, Nikolaos S. Thomaidis**

Electronic Supplementary Material

Table of contents

Table S1. Target list of phenolic compounds .... **Page 3**
Table S2. Validation Data of target screening methodology .... **Page 6**
Table S3 Mass features revealing pomegranate adulteration with apple in different adulteration levels .... **Page 8**
Table S4 Mass features revealing pomegranate adulteration with grape in different adulteration levels .... **Page 11**
Figure S1 EICs and MS spectra of caffeic acid in authentic and adulterated Ermioni pomegranate juices .... **Page 13**
Figure S2 EICs and MS spectra of catechin and epicatechin in authentic and adulterated pomegranate juices. .... **Page 14**
Figure S3 EICs and MS spectra of hydroxytyrosol and resveratrol in authentic and adulterated pomegranate juices. .... **Page 15**
Figure S4 Identification data for the mass feature m/z 193.0509_6.1 min (vanillin acetate) .... **Page 16**
Figure S5 Identification data for the mass feature m/z 273.0769_5.9 min (phloridzin in-source fragment) .... **Page 17**
Figure S6 Identification data for the mass features m/z 337.0943_3.9 min and m/z 337.0943_3.3 min (p-coumaroylquinic acid isomers) .... **Page 19**
Figure S7 Identification data for the mass feature m/z 191.0551_1.3 min (quinic acid) .... **Page 21**
Figure S8 Identification data for the mass feature m/z 307.1762_6.3 min ((R)-1-O-b-D-glucopyranosyl-1,3-octanediol) .... **Page 22**
Figure S9 Identification data for the mass feature m/z 351.1309_3.9 min (2-O-acetyl-alpha-D-abequopyranosyl-(1->3)-alpha-D-mannopyranose) .... **Page 23**
Figure S10 Identification data for the mass feature m/z 517.2284 _4.5 min (Vomifoliol 9-[xylosyl-(1->6)-glucoside]) .... **Page 24**
Figure S11 Identification data for the mass feature m/z 289.0830 _2.8 min (N2-malonyl-D-tryptophan) .... **Page 25**
Figure S12 Identification data for the mass feature m/z 273.0766 _7.5 min (phloretin) .... **Page 26**
Figure S13 Identification data for the mass feature m/z 491.1191_4.7 min (malvidin-3-O-glucoside) .... **Page 28**
Figure S14 Identification data for the mass feature m/z 261.0403 _4.9 min (maclurin) .... **Page 29**
Figure S15 Identification data for the mass feature m/z 389.1242 _4.8 min (resveratrol 3-glucoside) .... **Page 30**
Figure S16 Identification data for the mass feature m/z 295.0464_1.7 min (cis-coutaric acid) .... **Page 31**
Figure S17 Identification data for the mass feature m/z 261.1344_3.0 min (phaseolic acid) .... **Page 32**
Figure S18 Identification data for the mass feature m/z 261.1344_3.0 min (procyanidin B) .... **Page 33**

Table S1. Target list of phenolic compounds

Compound	Molecular formula	$[M-H]^-$ (m/z)	t_R (min)	Fragments (m/z)	Fragment elemental formula
2',4'-Dihydroxychalcone	$C_{15}H_{12}O_3$	239.0714	10.10	119.0491	C_8H_7O
4-hydroxybenzoic acid	$C_7H_6O_3$	137.0244	1.40	93.0340	C_6H_5O
				65.0390	C_5H_5
8-Prenylnaringenin	$C_{20}H_{20}O_5$	339.1238	10.00	219.0657	$C_{12}H_{11}O_4$
				119.0497	C_8H_7O
Apigenin	$C_{15}H_{10}O_5$	269.0455	8.10	117.0340	C_8H_5O
				151.0031	$C_7H_3O_4$
Caffeic acid	$C_9H_8O_4$	179.0350	1.40	135.0446	$C_8H_7O_2$
				134.0346	$C_8H_6O_2$
Catechin	$C_{15}H_{14}O_6$	289.0718	3.80	203.0705	$C_{12}H_{11}O_3$
				123.0458	$C_7H_7O_2$
Chrysin	$C_{15}H_{10}O_4$	253.0506	9.70	209.0597	$C_{14}H_9O_2$
				143.0491	$C_{10}H_7O$
Cinnamic acid	$C_9H_8O_2$	147.0452	4.50	103.0548	C_8H_7
				147.0446	$C_9H_7O_2$
				146.8973	$C_8H_3O_3$
Epicatechin	$C_{15}H_{14}O_6$	289.0718	4.30	137.0244	$C_7H_5O_3$
				151.0401	$C_8H_5O_4$
Eriodictyol	$C_{15}H_{12}O_6$	287.0561	6.40	151.0037	$C_7H_3O_4$
				135.0452	$C_8H_7O_2$
Ethyl vanillin	$C_9H_{10}O_3$	165.0557	5.60	136.0181	$C_7H_4O_3$
				137.0233	$C_7H_5O_3$
				108.0219	$C_6H_4O_2$
Ferulic acid	$C_{10}H_{10}O_4$	193.0506	3.00	134.0373	$C_8H_6O_2$
				178.0272	$C_9H_6O_4$
Galangin	$C_{15}H_{10}O_5$	269.0455	10.00	213.0546	$C_{13}H_9O_3$
				169.0657	$C_{12}H_9O$

				197.0597	$C_{13}H_9O_2$
Gallic acid	$C_7H_6O_5$	169.0142	1.30	125.0244	$C_6H_5O_3$
				69.0344	C_4H_5O
				97.0295	$C_5H_5O_2$
Genistein	$C_{15}H_{10}O_5$	269.0455	7.50	133.0284	$C_8H_5O_2$
				225.0546	$C_{14}H_9O_3$
				159.044	$C_{10}H_7O_2$
Gentistic acid	$C_7H_6O_4$	153.0193	2.40	108.0215	$C_6H_4O_2$
				109.0278	$C_6H_5O_2$
Hesperetin	$C_{16}H_{14}O_6$	301.0718	7.40	151.0025	$C_7H_3O_4$
				195.9988	$C_8H_4O_6$
Hydroxytyrosol	$C_8H_{10}O_3$	153.0557	3.50	123.0452	$C_7H_7O_2$
Luteolin	$C_{15}H_{10}O_6$	285.0405	7.55	133.0295	$C_8H_5O_2$
				151.0037	$C_7H_3O_4$
Myricetin	$C_{15}H_{10}O_8$	317.0303	6.10	151.0031	$C_7H_3O_4$
				178.998	$C_8H_3O_5$
Naringenin	$C_{15}H_{12}O_5$	271.0612	7.20	119.0502	C_8H_7O
				151.0037	$C_7H_3O_4$
				177.0193	$C_9H_5O_4$
p-coumaric acid	$C_9H_8O_3$	163.0401	2.60	119.0502	C_8H_7O
				93.0344	C_6H_5O
Pinobanksin	$C_{15}H_{12}O_5$	271.0612	7.20	253.0495	$C_{15}H_9O_4$
				197.0597	$C_{13}H_9O_2$
				225.0546	$C_{14}H_9O_3$
Pinocembrin	$C_{15}H_{12}O_4$	255.0663	9.20	151.0025	$C_7H_3O_4$
				213.0546	$C_{13}H_9O_3$
Pinoresinol	$C_{20}H_{22}O_6$	357.1344	6.49	151.0399	$C_8H_7O_3$
Protocatechuic acid	$C_7H_6O_4$	153.0193	1.30	109.0290	$C_6H_5O_2$
				108.0218	$C_6H_4O_2$
Quercetin	$C_{15}H_{10}O_7$	301.0354	7.10	151.0036	$C_7H_3O_4$

				178.9959	$C_8H_3O_5$
				121.0288	$C_7H_5O_2$
Resveratrol	$C_{14}H_{12}O_3$	227.0714	5.80	143.0502	$C_{10}H_7O$
				185.0608	$C_{12}H_9O_2$
Rosmarinic acid	$C_{18}H_{16}O_8$	359.0772	4.30	161.0233	$C_9H_5O_3$
				197.0444	$C_9H_9O_5$
				179.0338	$C_9H_7O_4$
Rutin	$C_{27}H_{30}O_{16}$	609.1461	5.50	301.0345	$C_{15}H_9O_7$
				300.0274	$C_{15}H_8O_7$
Salicylic acid	$C_7H_6O_3$	137.0244	3.60	93.034	C_6H_5O
				65.0399	C_5H_5
Syringaldehyde	$C_9H_{10}O_4$	181.0506	4.70	151.0031	$C_7H_3O_4$
				123.0082	$C_6H_3O_3$
				166.0265	$C_8H_6O_4$
Syringic acid	$C_9H_{10}O_5$	197.0455	1.40	123.008	$C_6H_3O_3$
				166.9976	$C_7H_3O_5$
Taxifolin	$C_{15}H_{12}O_7$	303.051	4.80	125.0227	$C_6H_5O_3$
				285.0408	$C_{15}H_9O_6$
				153.0193	$C_7H_5O_4$
Tyrosol	$C_8H_{10}O_2$	137.0608	4.10	119.0495	C_8H_7O
				107.0496	C_7H_7O
				93.034	C_6H_5O
Vanillic acid	$C_8H_8O_4$	167.035	1.40	125.0244	$C_6H_5O_3$
Vanillin	$C_8H_8O_3$	151.0401	4.70	136.0158	$C_7H_4O_3$

Table S2. Validation Data of target screening methodology

Analyte	Trueness<br>% Recovery	Repeatability<br>%RSD (n=6)	%Matrix Effect	MLOD (μg/L)	MLOQ (μg/L)
2',4'-Dihydroxychalcone	103	2.10	30	70.0	210.0
4-hydroxybenzoic acid	83.2	3.94	14	75.0	225.0
8-Prenylnaringenin	89.8	4.53	18	50.0	150.0
Apigenin	61.6	1.59	-23	5.4	16.1
Caffeic acid	90.1	2.11	-6	12.7	38.1
Catechin	87.9	1.15	-31	86.8	260.5
Chrysin	67.3	0.92	-1	7.4	22.2
Cinnamic acid	83.7	5.95	-19	55.0	165.0
Epicatechin	81.6	1.23	-62	11.5	34.4
Eriodictyol	58.8	4.27	-37	33.0	99.0
Ethyl vanillin	90.4	6.30	12	47.1	141.3
Ferulic acid	81.2	1.93	-23	55.0	165.0
Galangin	78.1	4.68	32	25.3	75.9
Gallic acid	61.8	1.27	-62	11.8	35.4
Genistein	60.1	1.18	-24	12.9	38.7
Gentistic acid	84.8	3.32	37	75.0	225.0
Hesperetin	77.9	3.80	0	9.5	28.5
Hydroxytyrosol	86.4	3.66	-23	106.3	318.8
Luteolin	55.3	5.08	11	17.5	52.5
Myricetin	63.9	3.65	80	34.4	103.1
Naringenin	82.6	1.64	-15	10.0	30.0
p-coumaric acid	68.1	5.19	-100	68.8	206.3
Pinobanksin	94.7	1.60	-16	8.3	24.9
Pinocembrin	75.3	1.35	-12	25.0	75.0
Pinoresinol	76.2	7.02	-10	21.2	63.5
Protocatechuic acid	72.3	1.41	-32	25.8	77.3

Quercetin	59.8	4.03	10	5.7	17.1
Resveratrol	91.1	6.11	-3	25.0	75.0
Rosmarinic acid	79.9	2.11	31	20.1	60.4
Rutin	69.8	4.97	12	80.0	240.0
Salicylic acid	92.3	7.25	77	29.7	89.1
Syringaldehyde	82.3	4.41	4	20.5	61.5
Syringic acid	81.4	4.60	18	48.5	145.6
Taxifolin	82.8	5.63	-43	7.2	21.7
Tyrosol	82.3	4.55	-19	27.0	81.1
Vanillic acid	59.7	2.30	37	91.7	275.0
Vanillin	85.3	1.70	111	103.1	309.4

Table S3: Mass features revealing **pomegranate** adulteration with **apple** in different adulteration levels.

m/z	Retention time (min)	20% adulteration	10% adulteration	5% adulteration	3% adulteration	2% adulteration	1% adulteration
191.0564	2.9	✓	✓	✓	✓	✓	✓
353.0877	2.9	✓	✓	✓	✓	✓	✓
193.0509	6.1	✓	✓	✓	✓	✓	✓
337.0942	3.9	✓	✓	✓	✓	✓	✗
353.088	3.4	✓	✓	✓	✓	✓	✗
338.0963	3.9	✓	✓	✓	✓	✓	✗
183.0664	3.7	✓	✓	✓	✓	✓	✗
351.1307	3.9	✓	✓	✓	✓	✗	✗
273.0769	5.9	✓	✓	✓	✓	✗	✗
307.1763	6.3	✓	✓	✓	✓	✗	✗
173.0458	3.9	✓	✓	✓	✓	✗	✗
192.0596	2.9	✓	✓	✓	✓	✗	✗
191.0568	1.3	✓	✓	✓	✓	✗	✗
161.0819	2.1	✓	✓	✓	✓	✗	✗
563.235	4.5	✓	✓	✓	✗	✗	✗
707.1808	2.9	✓	✓	✓	✗	✗	✗
245.0935	2.8	✓	✓	✓	✗	✗	✗
289.0832	2.8	✓	✓	✓	✗	✗	✗
405.1767	3.5	✓	✓	✓	✗	✗	✗
471.1057	5.9	✓	✓	✓	✗	✗	✗
473.1048	5.9	✓	✓	✓	✗	✗	✗
517.2289	4.5	✓	✓	✓	✗	✗	✗
498.1246	5.9	✓	✓	✓	✗	✗	✗
337.0942	3.4	✓	✓	✓	✗	✗	✗
165.0777	1.5	✓	✓	✓	✗	✗	✗
393.1768	5	✓	✓	✓	✗	✗	✗
195.0882	1.5	✓	✓	✓	✗	✗	✗
425.1663	3.9	✓	✓	✓	✗	✗	✗

351.1299	3.6	✓	✓	✕	✕	✕	✕
451.1243	5.3	✓	✓	✕	✕	✕	✕
497.2234	5.1	✓	✓	✕	✕	✕	✕
307.1763	6	✓	✓	✕	✕	✕	✕
96.009	2.6	✓	✓	✕	✕	✕	✕
437.2023	5.1	✓	✓	✕	✕	✕	✕
456.151	4.1	✓	✓	✕	✕	✕	✕
517.3162	7.4	✓	✓	✕	✕	✕	✕
447.0952	6.0	✓	✓	✕	✕	✕	✕
191.0565	3.4	✓	✓	✕	✕	✕	✕
429.1769	3.6	✓	✓	✕	✕	✕	✕
469.2284	5.8	✓	✓	✕	✕	✕	✕
577.2506	4.5	✓	✓	✕	✕	✕	✕
179.0352	2.9	✓	✓	✕	✕	✕	✕
510.0888	6.0	✓	✓	✕	✕	✕	✕
568.1744	5.5	✓	✓	✕	✕	✕	✕
567.172	5.5	✓	✓	✕	✕	✕	✕
439.218	6.0	✓	✓	✕	✕	✕	✕
518.2318	4.5	✓	✓	✕	✕	✕	✕
501.3215	10.6	✓	✓	✕	✕	✕	✕
413.1306	1.5	✓	✕	✕	✕	✕	✕
273.0769	7.5	✓	✕	✕	✕	✕	✕
475.1313	1.4	✓	✕	✕	✕	✕	✕
728.2276	3.4	✓	✕	✕	✕	✕	✕
485.2236	6.0	✓	✕	✕	✕	✕	✕
609.1946	3.3	✓	✕	✕	✕	✕	✕
93.0345	2.9	✓	✕	✕	✕	✕	✕
467.1191	2.0	✓	✕	✕	✕	✕	✕
597.1814	5.2	✓	✕	✕	✕	✕	✕
289.0718	5.3	✓	✕	✕	✕	✕	✕
588.1902	3.4	✓	✕	✕	✕	✕	✕
579.1475	1.0	✓	✕	✕	✕	✕	✕
207.0652	7.0	✓	✕	✕	✕	✕	✕
481.1341	5.8	✓	✕	✕	✕	✕	✕

425.2025	5.7	✓	✕	✕	✕	✕	✕
463.0883	5.5	✓	✕	✕	✕	✕	✕
446.0816	1.0	✓	✕	✕	✕	✕	✕
580.2253	4.5	✓	✕	✕	✕	✕	✕
337.114	3.1	✓	✕	✕	✕	✕	✕
**Total Markers**		**67**	**48**	**28**	**14**	**7**	**3**

Table S4: Mass features revealing **pomegranate** adulteration with **red grape** in different adulteration levels.

m/z	Retention time (min)	20% adulteration	10% adulteration	5% adulteration	3% adulteration	2% adulteration	1% adulteration
369.0288	2.2	✓	✓	✓	✓	✓	✓
149.0096	1.2	✓	✓	✓	✓	✓	✓
287.1502	4.0	✓	✓	✓	✓	✓	✓
491.1191	4.7	✓	✓	✓	✓	✓	✗
295.0464	1.7	✓	✓	✓	✓	✗	✗
261.0405	4.9	✓	✓	✓	✓	✗	✗
389.1242	4.8	✓	✓	✓	✓	✗	✗
283.0396	2.7	✓	✓	✓	✓	✗	✗
311.0808	2.3	✓	✓	✓	✓	✗	✗
261.1344	3.0	✓	✓	✓	✓	✗	✗
477.0671	5.0	✓	✓	✓	✗	✗	✗
369.0288	3.2	✓	✓	✓	✗	✗	✗
509.1298	3.3	✓	✓	✓	✗	✗	✗
167.0348	5.5	✓	✓	✓	✗	✗	✗
427.0340	4.1	✓	✓	✓	✗	✗	✗
577.1346	3.4	✓	✓	✓	✗	✗	✗
295.0858	3.8	✓	✓	✓	✗	✗	✗
429.2132	3.4	✓	✓	✗	✗	✗	✗
121.0293	2.6	✓	✓	✗	✗	✗	✗
190.0541	2.8	✓	✓	✗	✗	✗	✗
315.0725	2.0	✓	✓	✗	✗	✗	✗
203.1076	6.6	✓	✓	✗	✗	✗	✗
293.1030	6.6	✓	✓	✗	✗	✗	✗
161.0818	2.1	✓	✓	✗	✗	✗	✗
397.0235	4.1	✓	✓	✗	✗	✗	✗
295.0857	4.5	✓	✓	✗	✗	✗	✗
461.1088	4.6	✓	✓	✗	✗	✗	✗
231.1027	5.4	✓	✓	✗	✗	✗	✗

219.1027	6.6	✓	✓	✗	✗	✗	✗
637.1555	6.9	✓	✓	✗	✗	✗	✗
423.0720	7.9	✓	✓	✗	✗	✗	✗
591.1022	2.0	✓	✓	✗	✗	✗	✗
446.0759	2.4	✓	✓	✗	✗	✗	✗
369.0288	3.0	✓	✓	✗	✗	✗	✗
577.1346	3.8	✓	✓	✗	✗	✗	✗
305.0303	3.8	✓	✓	✗	✗	✗	✗
209.0304	1.1	✓	✓	✗	✗	✗	✗
449.1087	5.3	✓	✗	✗	✗	✗	✗
163.0401	1.7	✓	✗	✗	✗	✗	✗
131.0712	3.1	✓	✗	✗	✗	✗	✗
330.2037	3.3	✓	✗	✗	✗	✗	✗
366.1198	3.5	✓	✗	✗	✗	✗	✗
107.0502	3.7	✓	✗	✗	✗	✗	✗
373.1143	3.2	✓	✗	✗	✗	✗	✗
187.0974	3.3	✓	✗	✗	✗	✗	✗
243.1239	3.6	✓	✗	✗	✗	✗	✗
413.2403	5.3	✓	✗	✗	✗	✗	✗
**Total Markers**		**47**	**37**	**17**	**10**	**4**	**3**

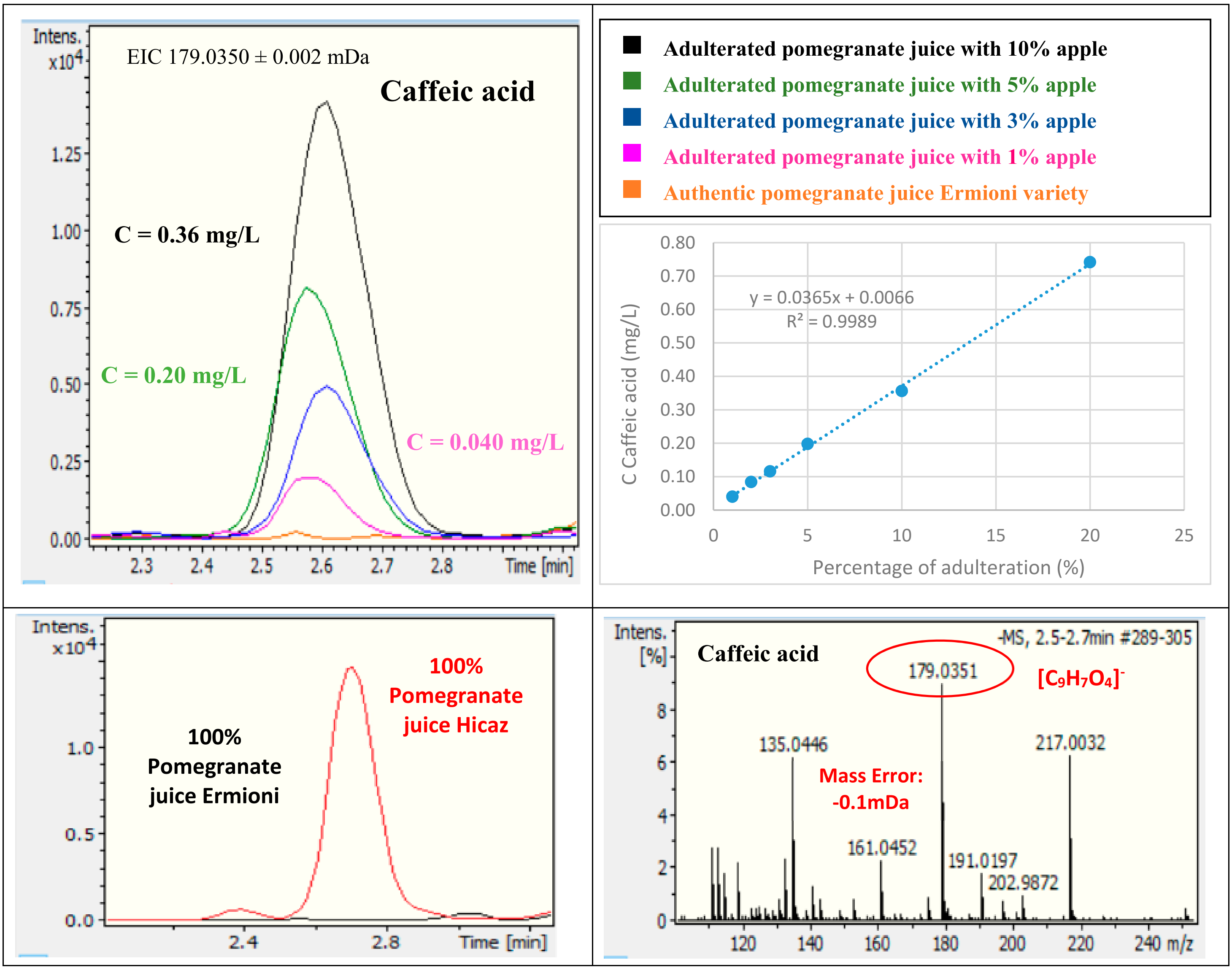

Figure S1. EICs and MS spectra of caffeic acid in authentic and adulterated Ermioni pomegranate juices.

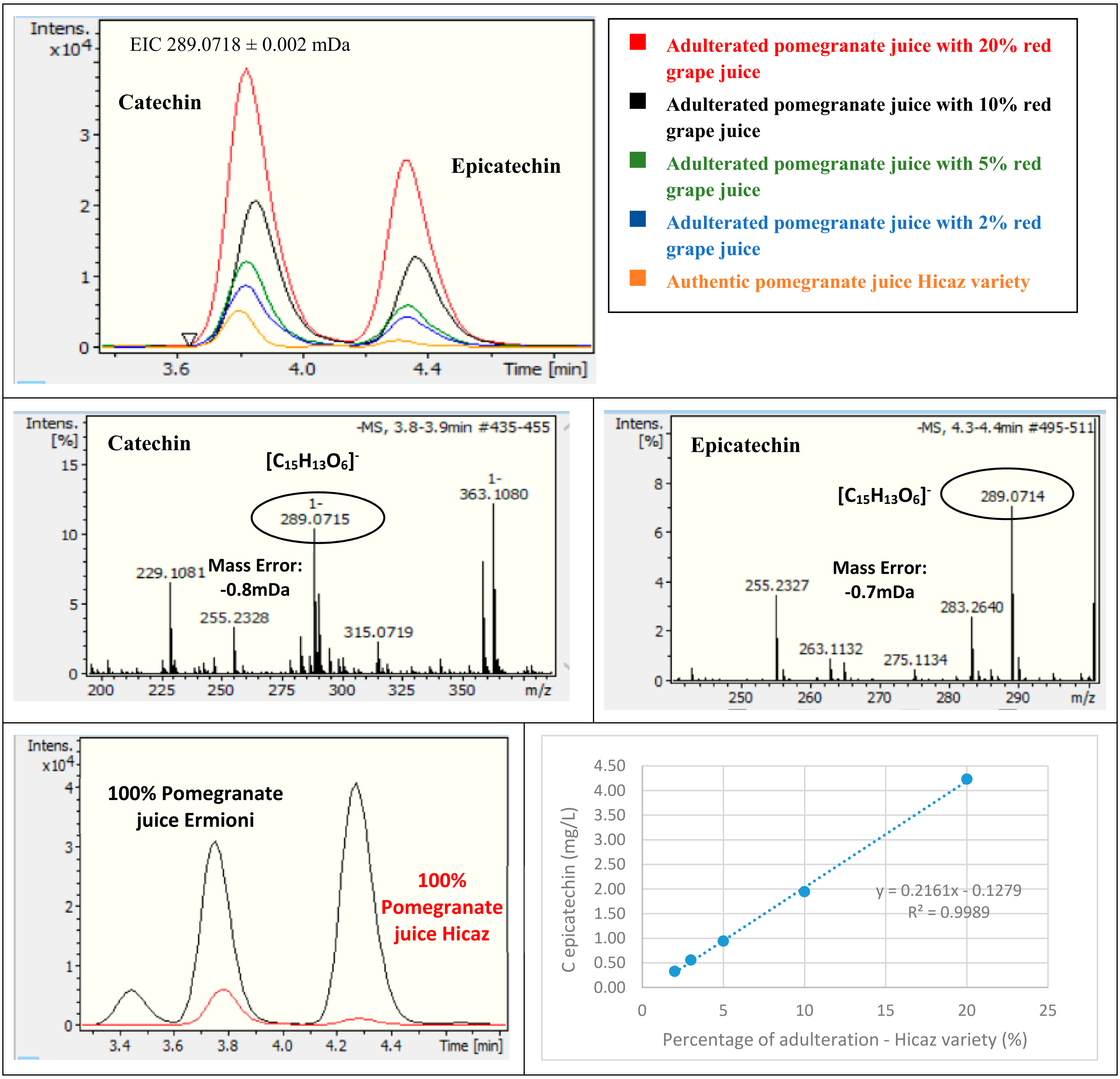

Figure S2. EICs and MS spectra of catechin and epicatechin in authentic and adulterated pomegranate juices.

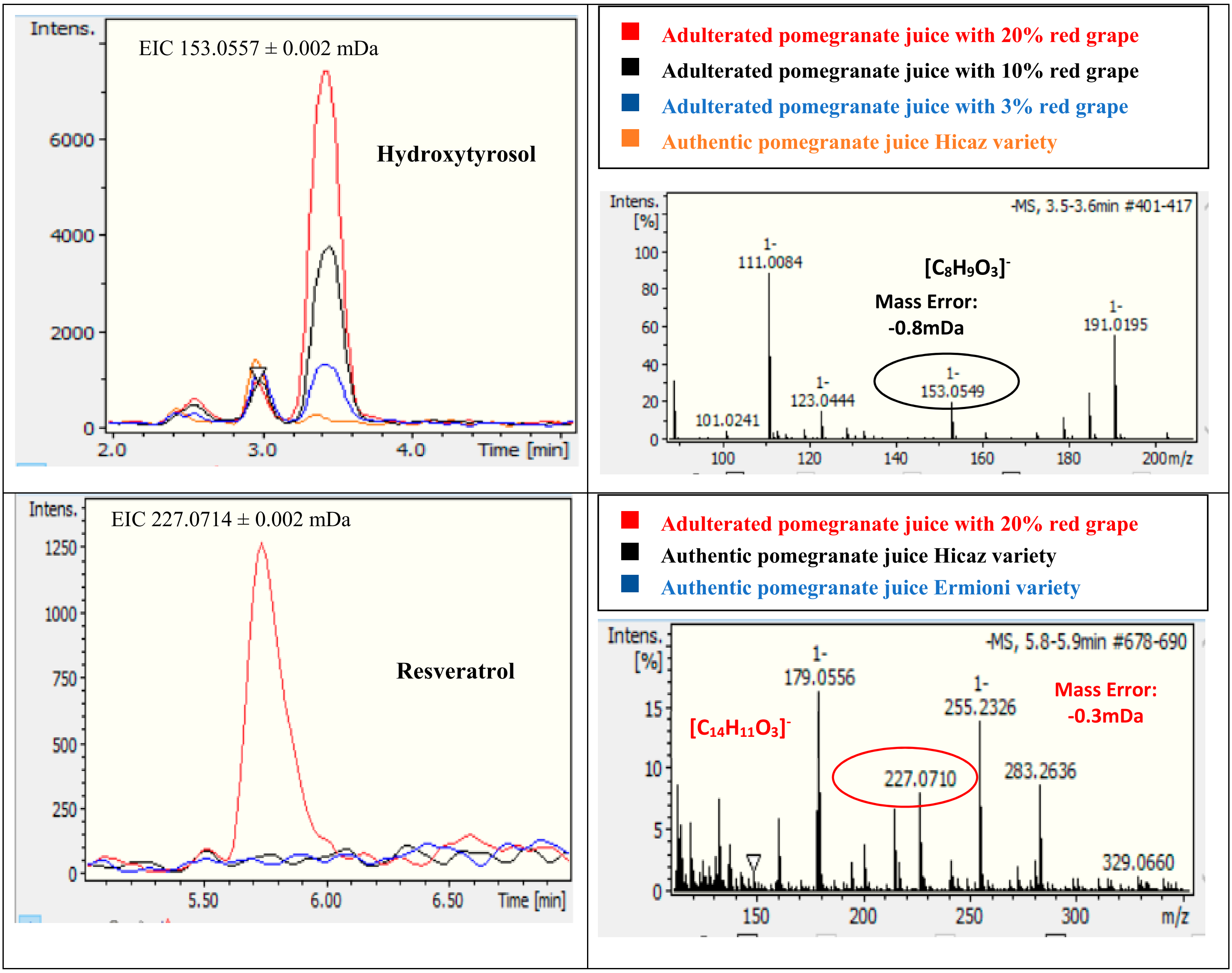

Figure S3. EICs and MS spectra of hydroxytyrosol and resveratrol in authentic and adulterated pomegranate juices.

Tentative identification of characteristic markers of apple juice obtained from untargeted workflow

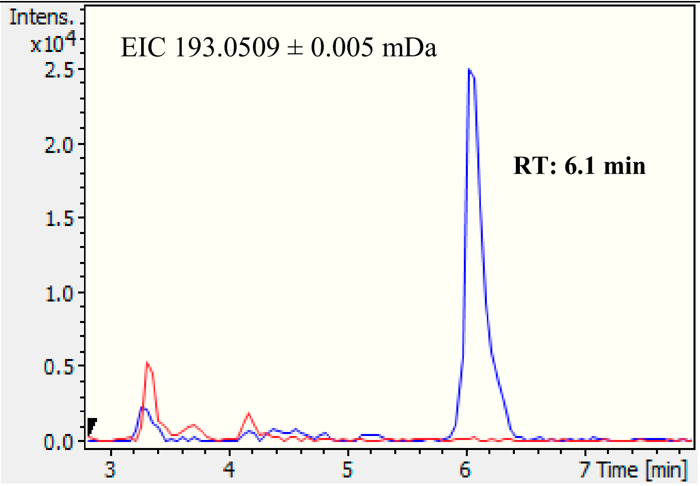

Fig S4a. EIC of m/z 193.0509 in **pomegranate - apple juice**

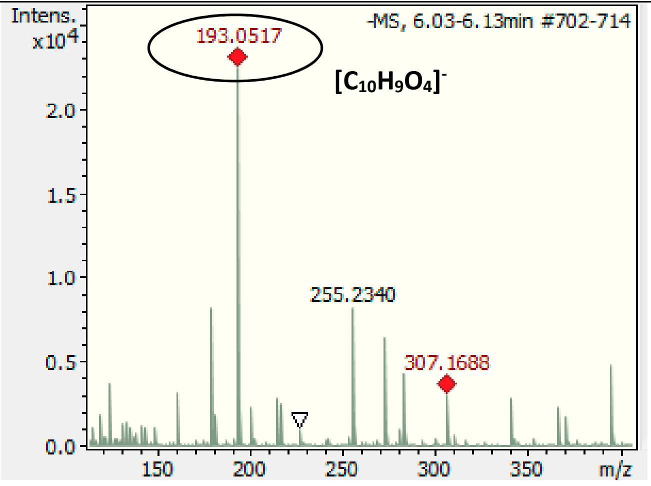

Fig S4b. MS Spectra of mass feature **m/z 193.0509_6.1 min**

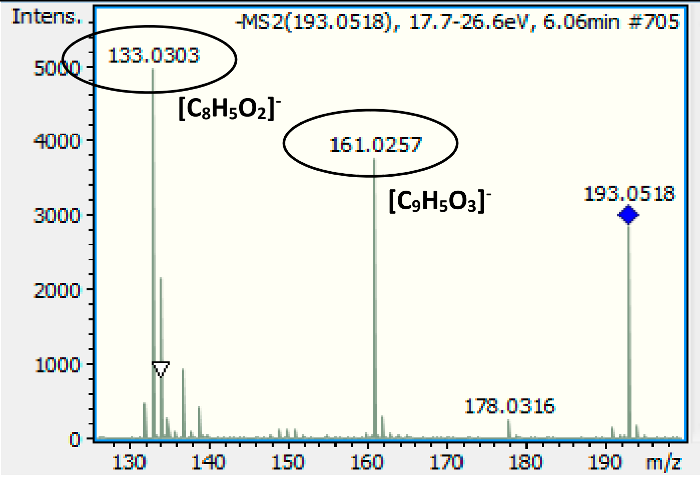

Fig S4c. MS/MS Spectra of mass feature **m/z 193.0509_6.1 min**

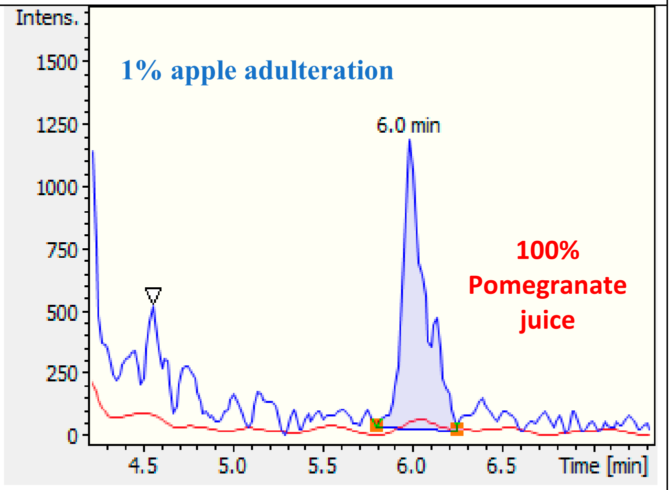

Fig S4d. EIC of m/z 193.0509 in authentic and adulterated pomegranate juice

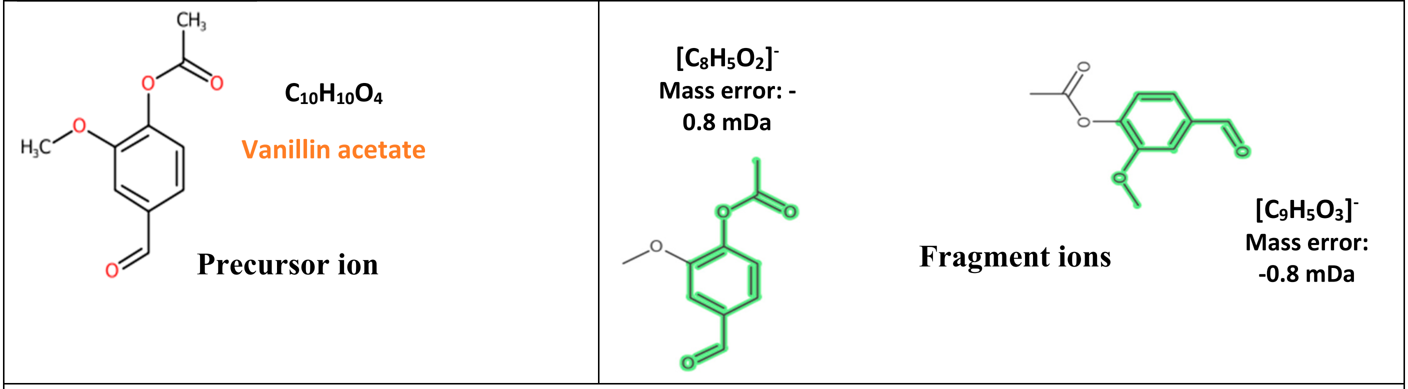

Fig S4e. Structures of precursor and fragment ions of vanillin acetate

Figure S4. Identification data for the mass feature **m/z 193.0509_6.1 min** (vanillin acetate)

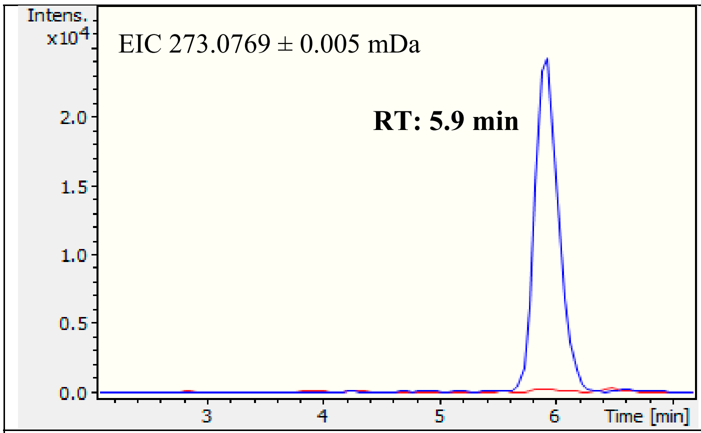

Fig S5a. EIC of m/z 273.0769 in **pomegranate - apple juice**

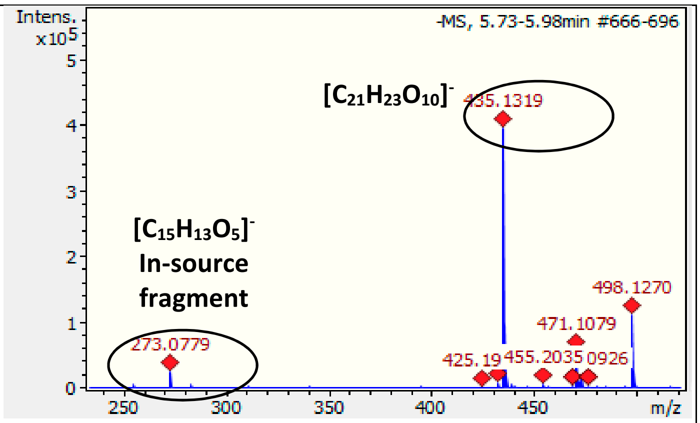

Fig S5b. MS spectra of mass feature **m/z 273.0769_ 5.9 min** in apple juice

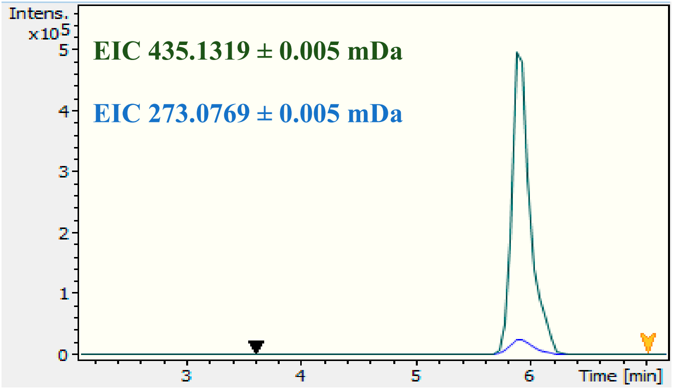

Fig S5c. EIC of precursor ion and in-source fragment in apple juice

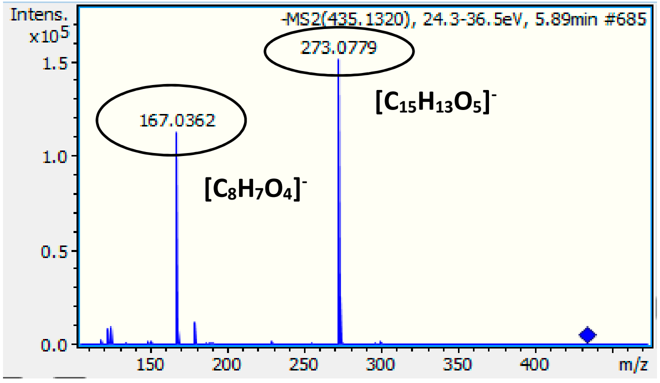

Fig S5d. MS/MS spectra of mass feature **m/z 435.1319_ 5.9 min** in apple juice

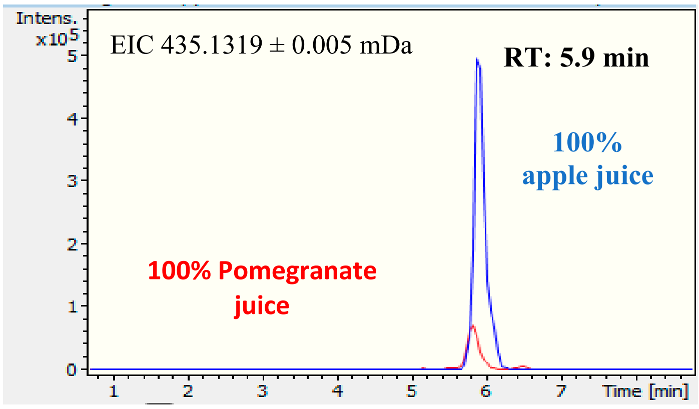

Fig S5e. EIC of m/z 435.1319 in pomegranate juice - apple juice

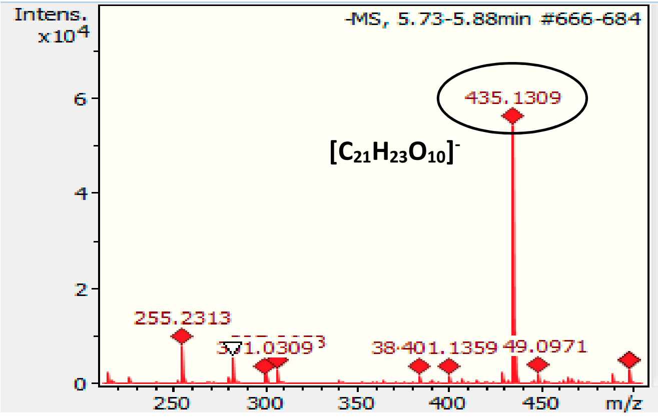

Fig S5f. MS spectra of mass feature **m/z 435.1319_ 5.9 min** in pomegranate juice

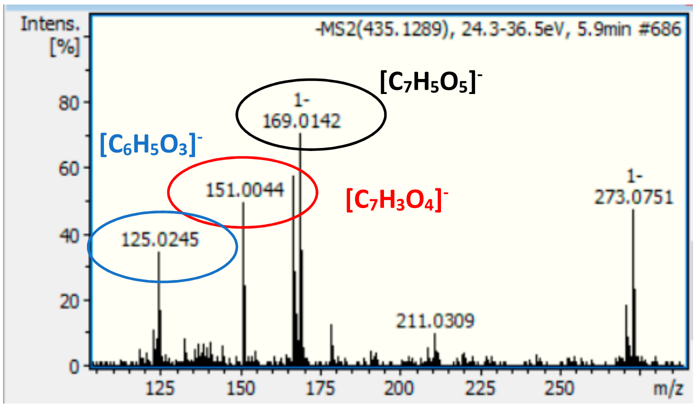

Fig S5g. MS/MS spectra of mass feature **m/z 273.0771_ 5.9 min** in pomegranate juice

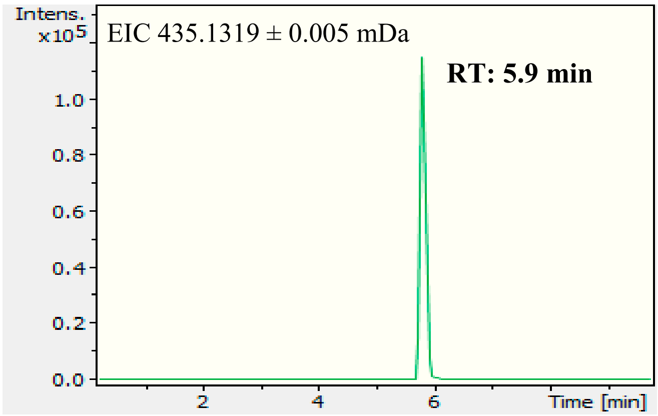

Fig S5h. EIC of m/z 435.1319 in phloridzin reference standard (C = 5 mg/L)

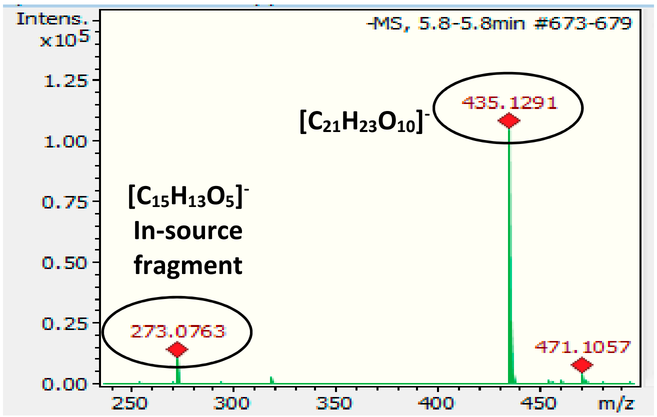

Fig S5i. MS spectra (phloridzin reference standard)

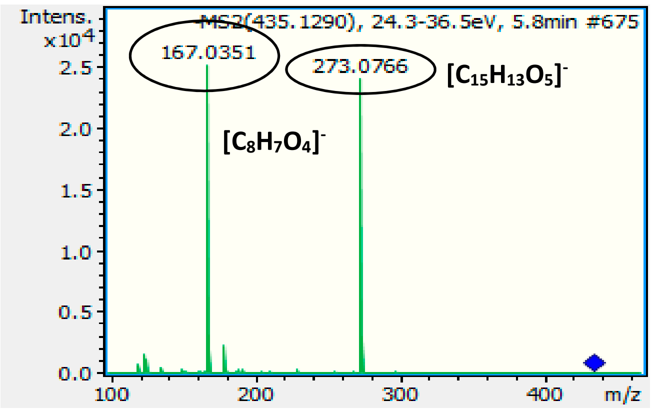

Fig S5j. MS/MS spectra (phloridzin reference standard)

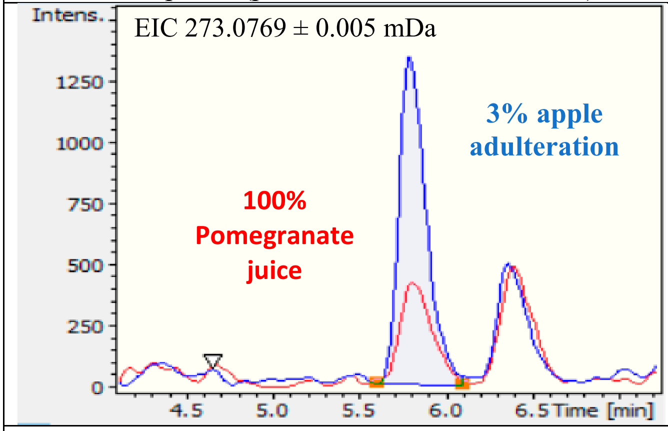

Fig S5k. EIC of m/z 273.0769 (in-source fragment of phloridzin) in authentic and adulterated pomegranate juice samples

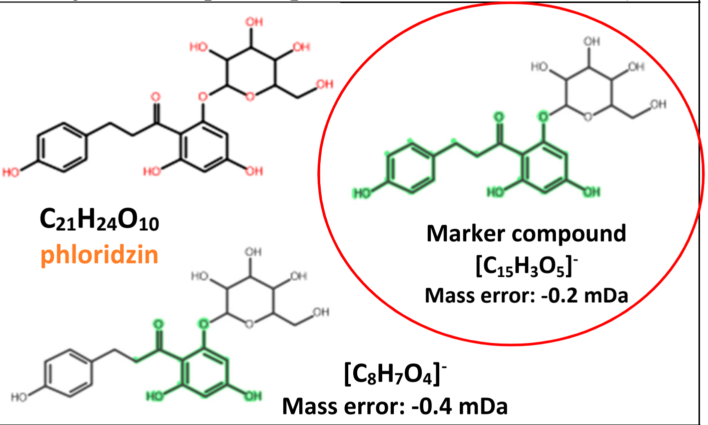

Fig S5l. Structures of precursor and fragment ions of phloridzin

Figure S5. Identification data for the mass feature **m/z 273.0769_5.9 min** (phloridzin in-source fragment).

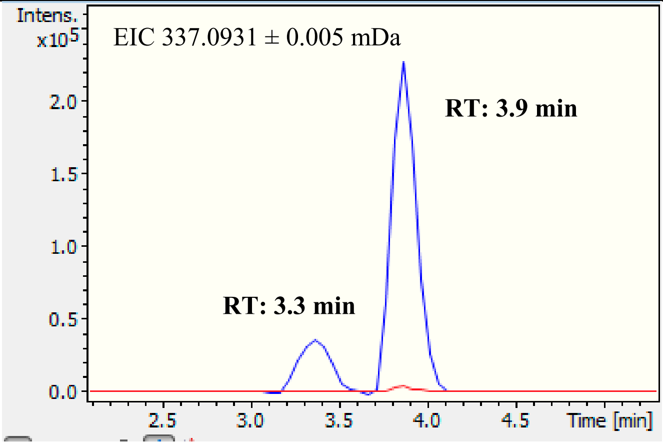

Fig S6a. EIC of m/z 337.0931 in pomegranate - apple juice

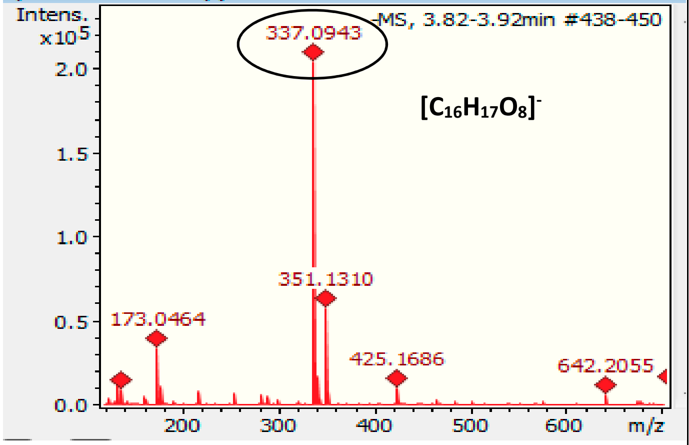

Fig S6b. MS Spectra of mass feature **m/z 337.0931_3.9 min**

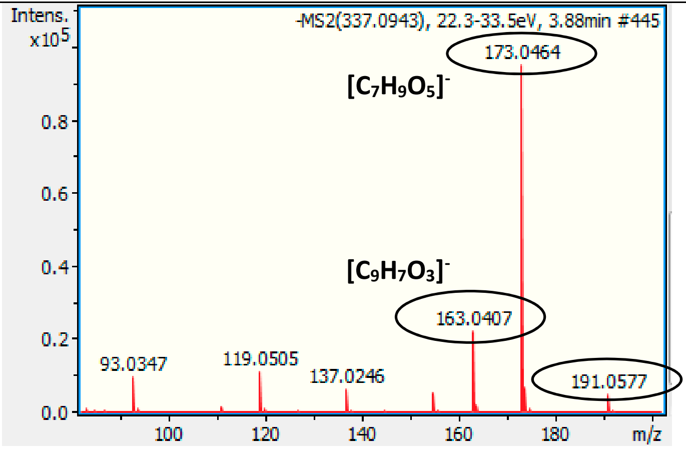

Fig S6c. MS/MS Spectra of mass feature **m/z 337.0931_3.9 min**

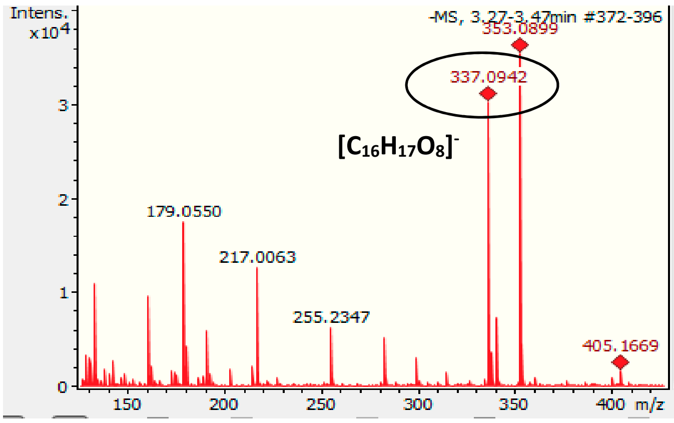

Fig S6d. MS Spectra of mass feature **m/z 337.0931_3.3 min**

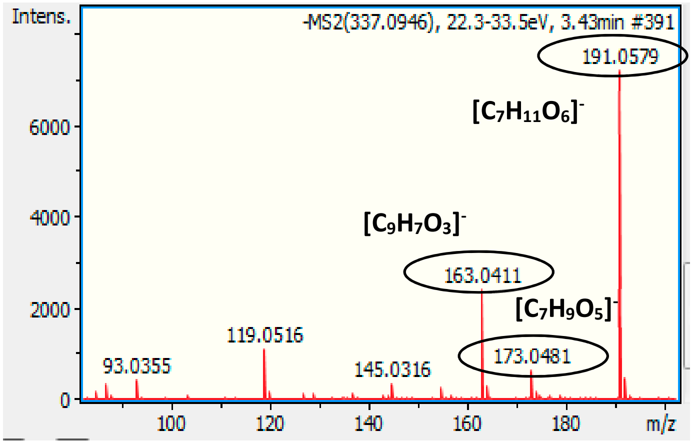

Fig S6e. MS/MS Spectra of mass feature **m/z 337.0931_3.3 min**

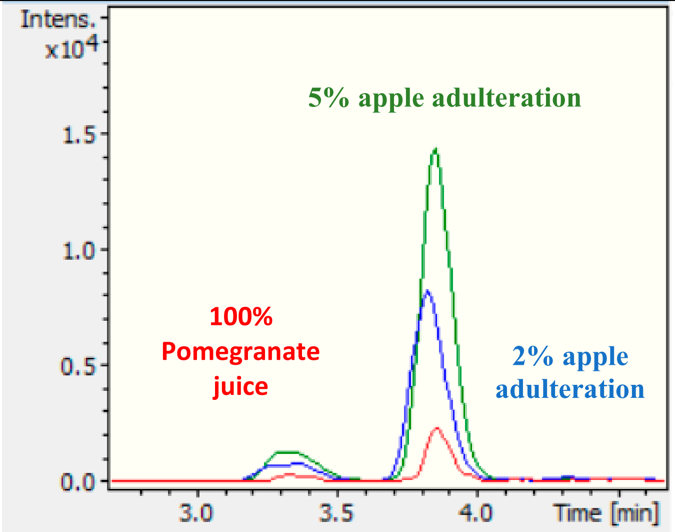

Fig S6f. EIC of m/z 337.0931 in authentic and adulterated pomegranate juice samples

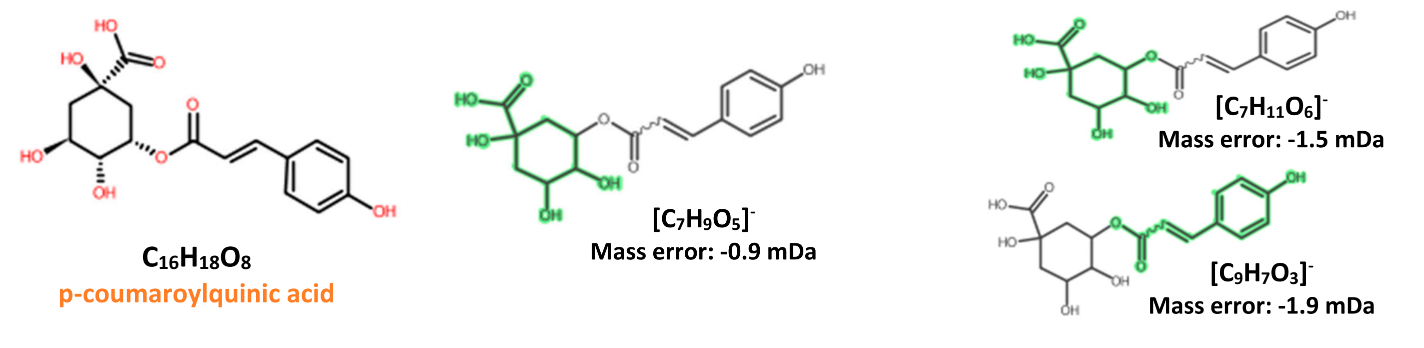

Fig S6g. Structures of precursor and fragment ions of p-coumaroylquinic acid

Figure S6. Identification data for the mass features **m/z 337.0943_3.9 min** and **m/z 337.0943_3.3 min** (p-coumaroylquinic acid isomers)

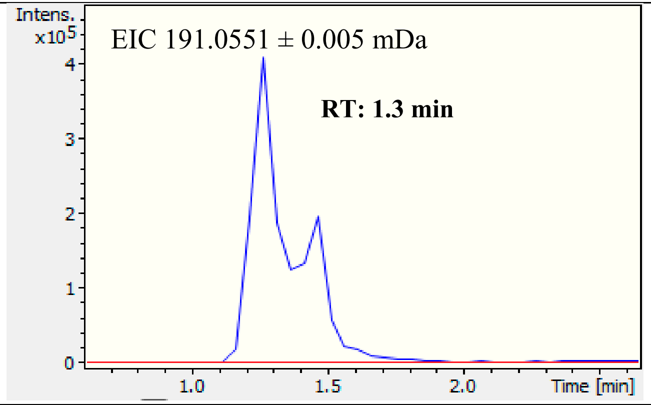

Fig S7a. EIC of m/z 191.0551 in pomegranate - apple juice

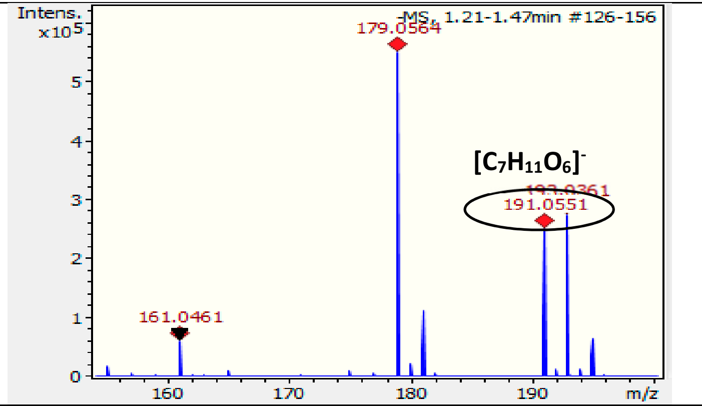

Fig S7b. MS Spectra of mass feature **m/z 191.0551_1.3 min**

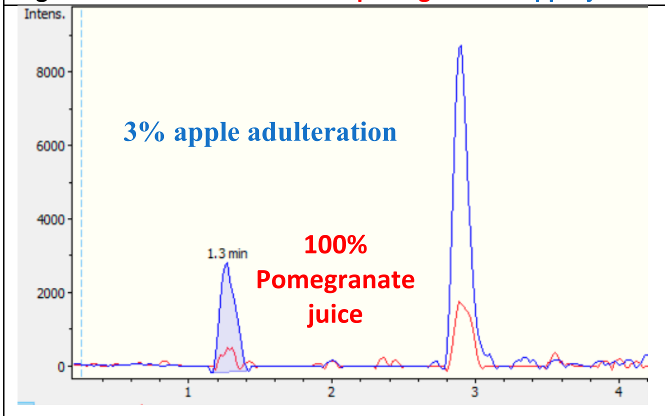

Fig S7c. EIC of m/z 191.0551 in authentic and adulterated pomegranate juice samples

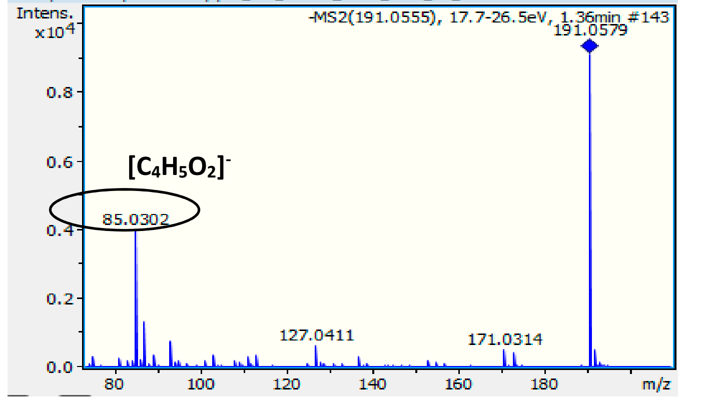

Fig S7d. MS/MS Spectra of mass feature **m/z 191.0551_1.3 min**

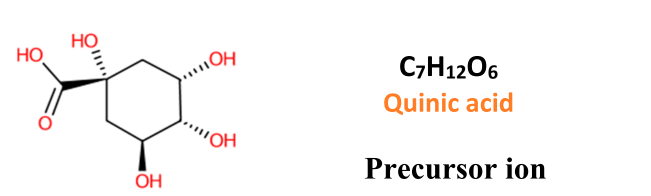

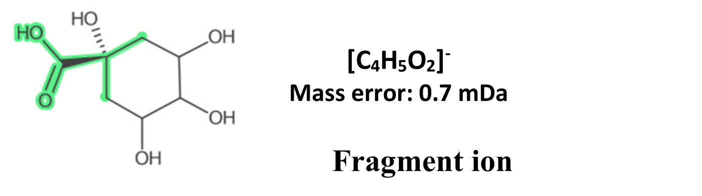

Fig S7e. Structures of precursor and fragment ions of quinic acid

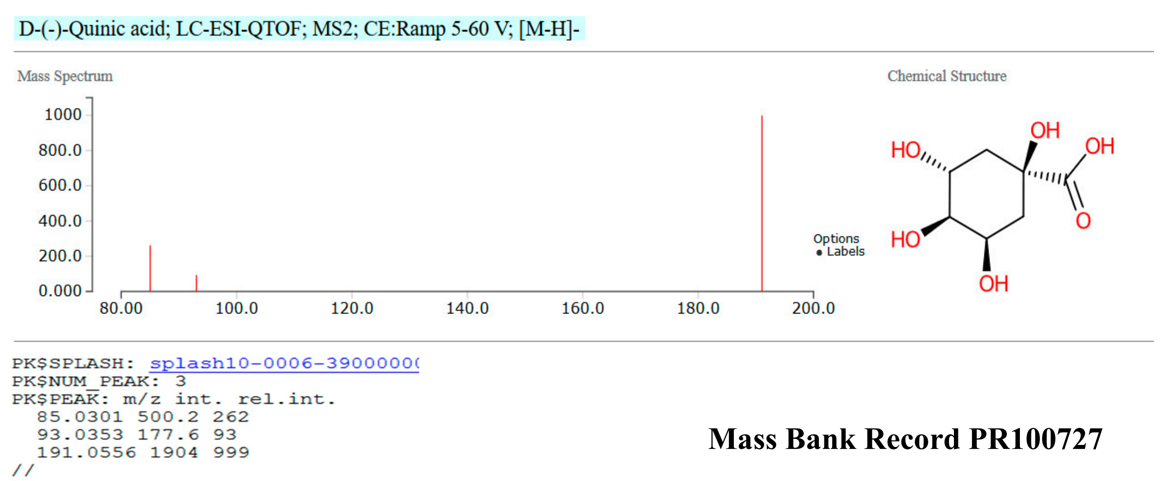

Fig. S7f: Mass Bank Record PR100727 (quinic acid)

Figure S7. Identification data for the mass feature **m/z 191.0551_1.3 min** (quinic acid).

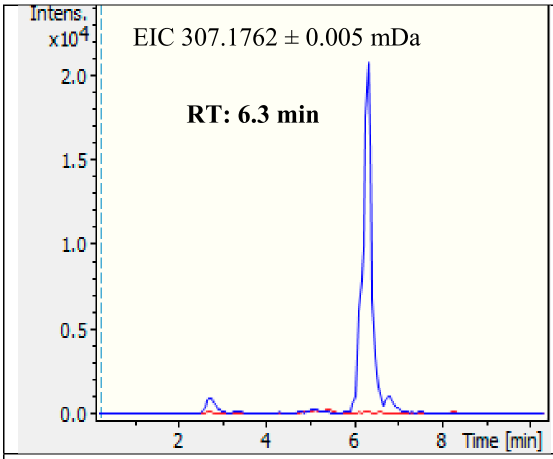

Fig S8a. EIC of m/z 307.1762 in pomegranate - apple juice

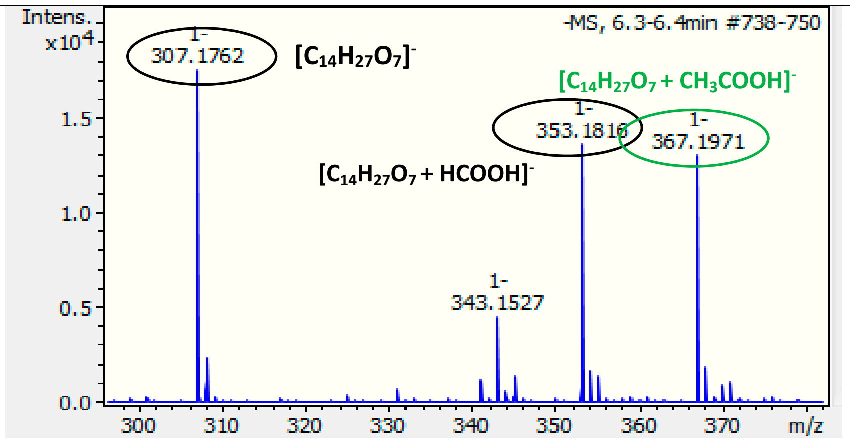

Fig S8b. MS Spectra of mass feature **m/z 307.1762_6.3 min**

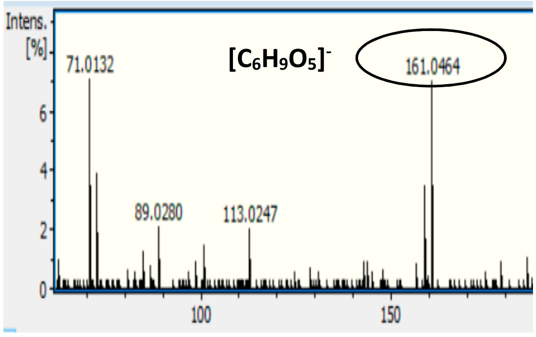

Fig S8c. MS/MS Spectra of mass feature **m/z 307.1762_6.3 min**

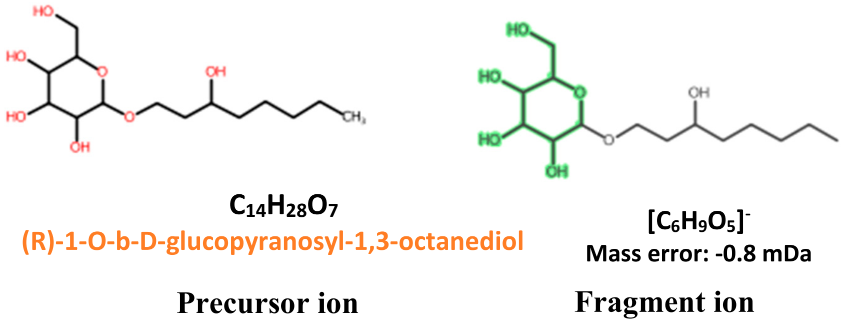

Fig S8d. Structures of precursor and fragment ions of (R)-1-O-b-D-glucopyranosyl-1,3-octanediol

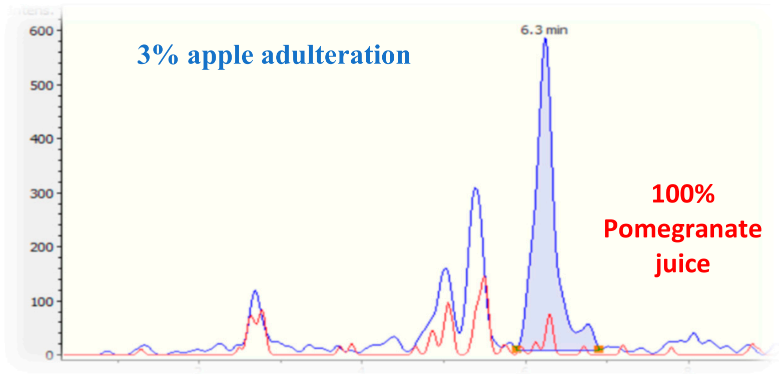

Fig S8e. EIC of m/z 307.1762 in authentic and adulterated pomegranate juice samples

Figure S8. Identification data for the mass feature **m/z 307.1762_6.3 min** ((R)-1-O-b-D-glucopyranosyl-1,3-octanediol).

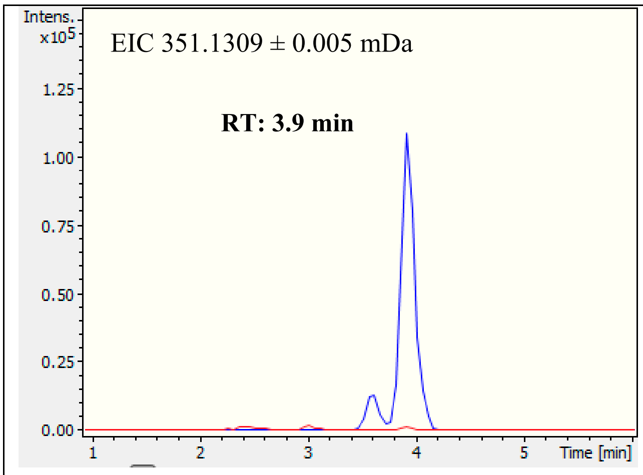

Fig S9a. EIC of m/z 351.1309 in **pomegranate - apple juice**

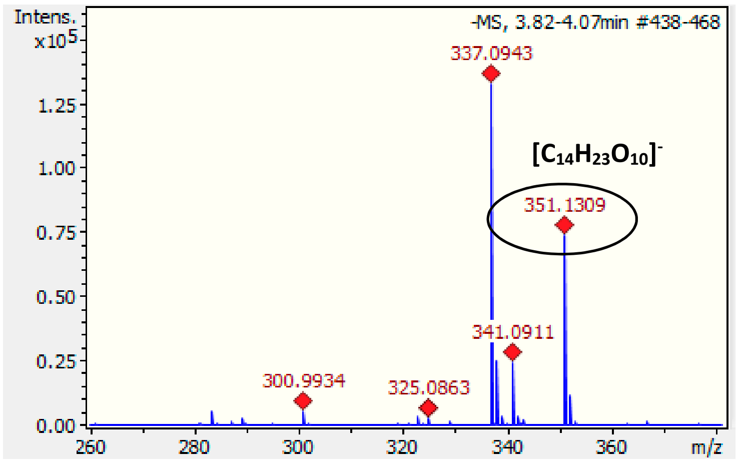

Fig S9b. MS Spectra of mass feature **m/z 351.1309_6.3 min**

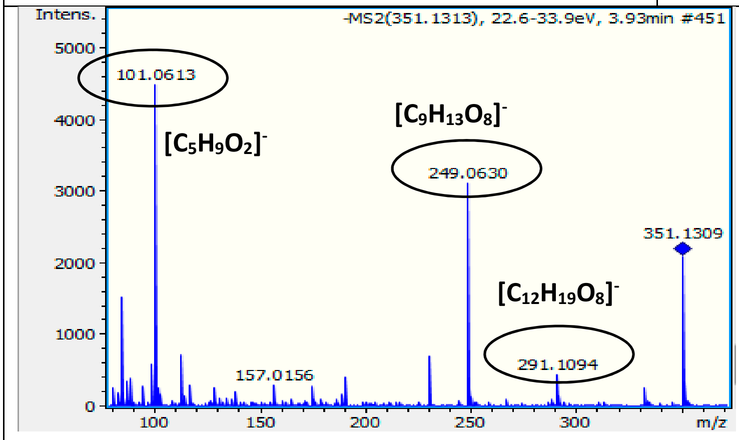

Fig S9c. MS/MS Spectra of mass feature **m/z 351.1309_6.3 min**

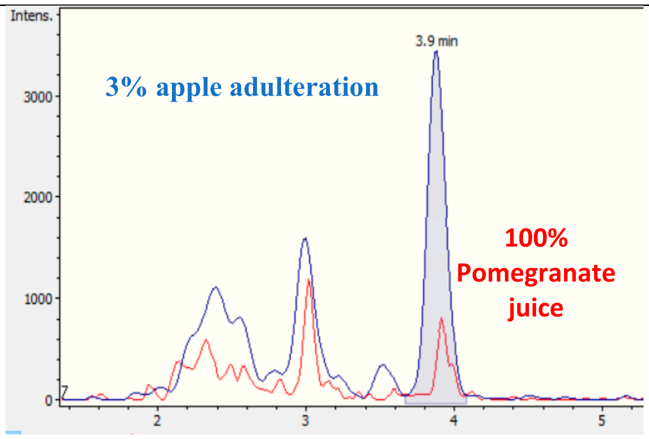

Fig S9d. EIC of m/z 351.1309 in authentic and adulterated pomegranate juice samples

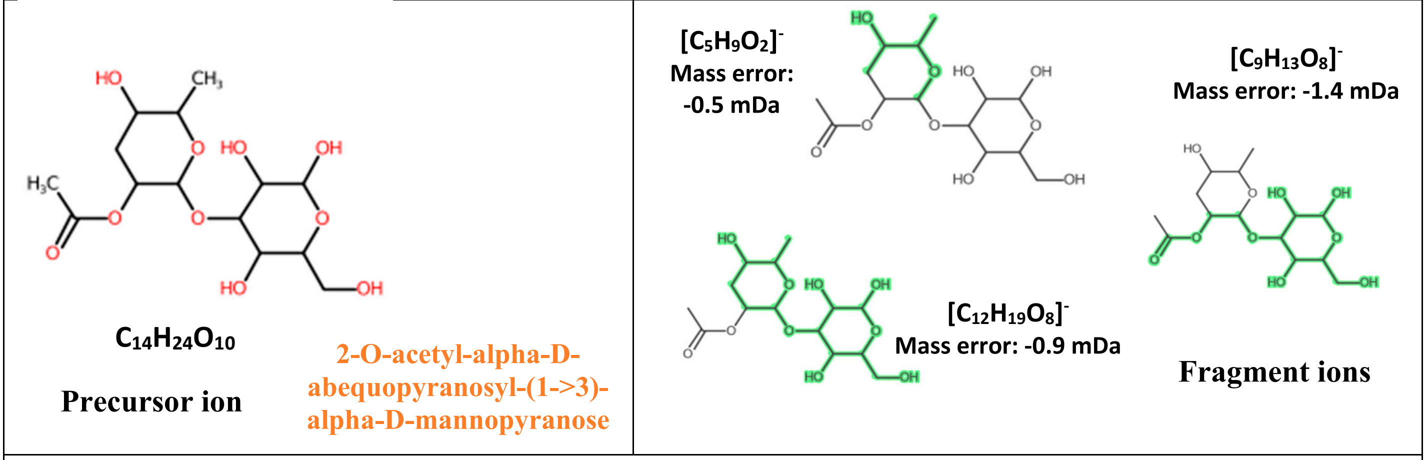

Fig S9e. Structures of precursor and fragment ions of 2-O-acetyl-alpha-D-abequopyranosyl-(1->3)-alpha-D-mannopyranose

Figure S9. Identification data for the mass feature **m/z 351.1309_3.9 min** (2-O-acetyl-alpha-D-abequopyranosyl-(1->3)-alpha-D-mannopyranose).

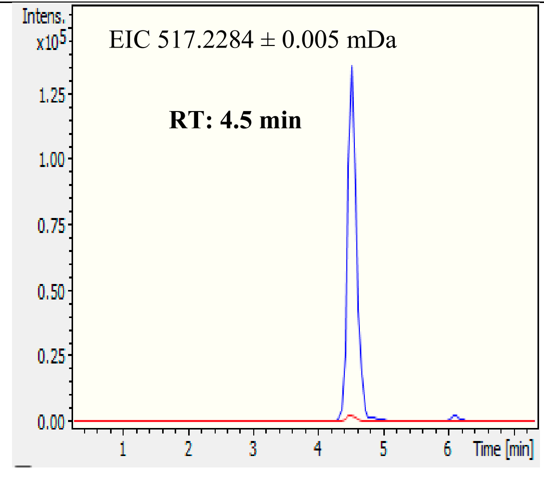

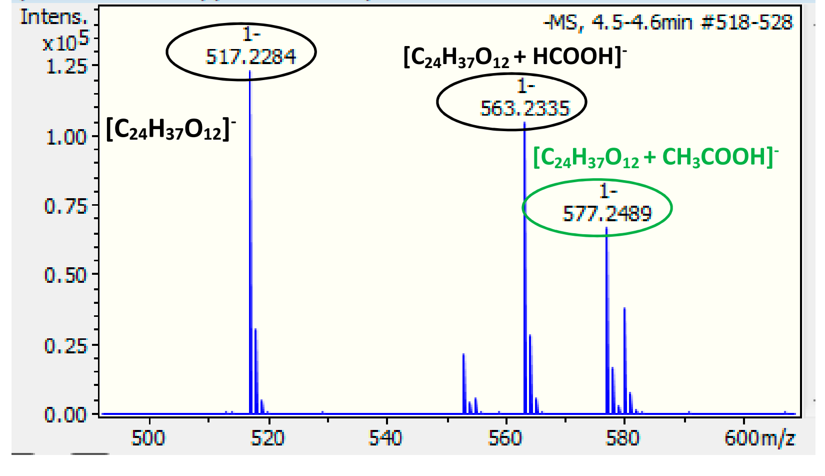

Fig S10a. EIC of m/z 517.2284 in **pomegranate - apple juice**

Fig S10b. MS Spectra of mass feature **m/z 517.2284_4.5 min**

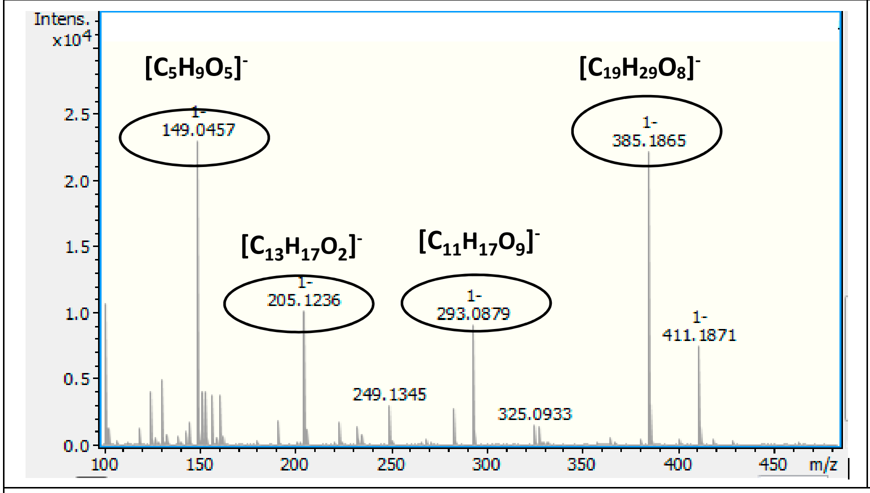

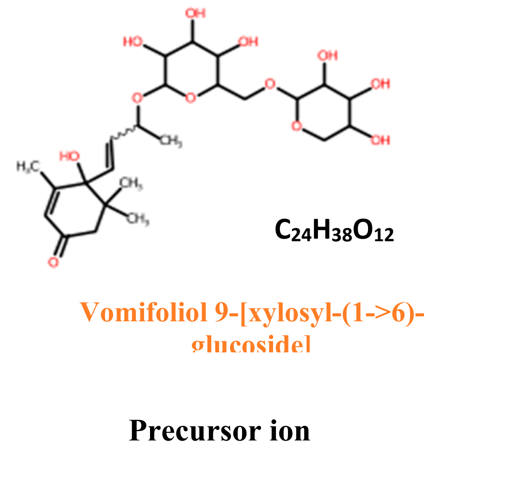

Fig S10c. MS/MS Spectra of mass feature **m/z 517.2284_4.5 min**

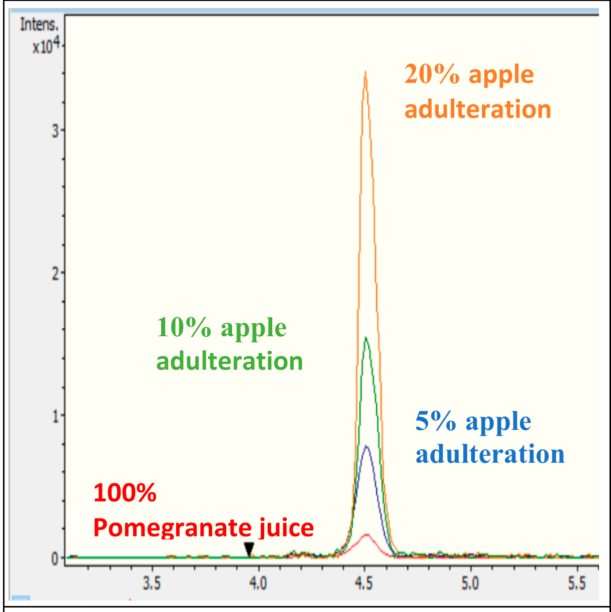

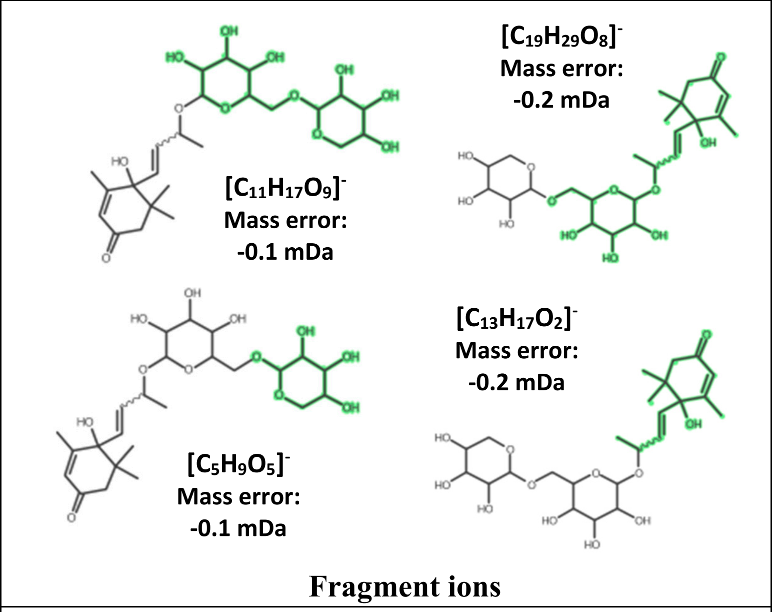

Fig S10d. EIC of m/z 517.2284 in authentic and adulterated pomegranate juice samples

Fig S10e. Structures of precursor and fragment ions of Vomifoliol 9-[xylosyl-(1->6)-glucoside]

Figure S10. Identification data for the mass feature **m/z 517.2284 _4.5 min** (Vomifoliol 9-[xylosyl-(1->6)-glucoside]).

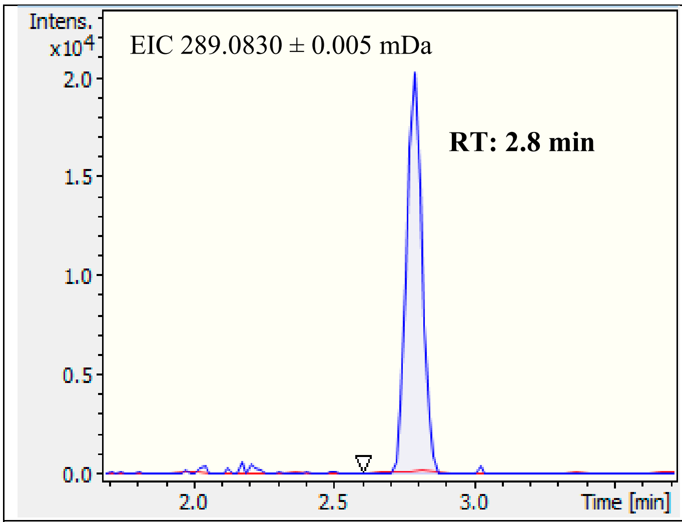

Fig S11a. EIC of m/z 289.0830 in **pomegranate - apple juice**

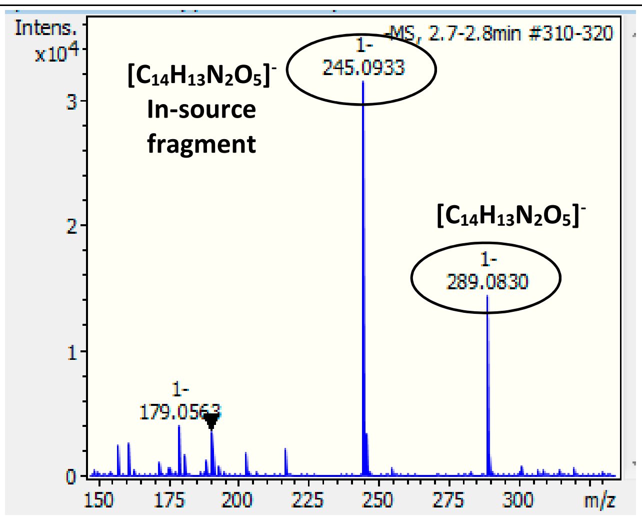

Fig S11b. MS Spectra of mass feature **m/z 289.0830_2.8 min**

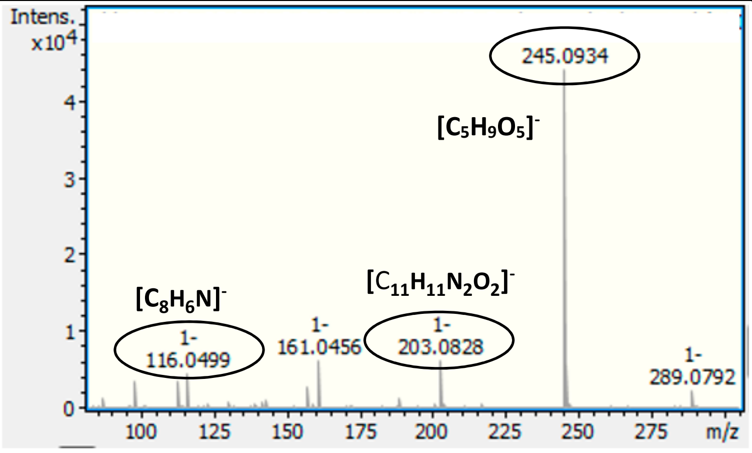

Fig S11c. MS Spectra of mass feature **m/z 289.0830_2.8 min**

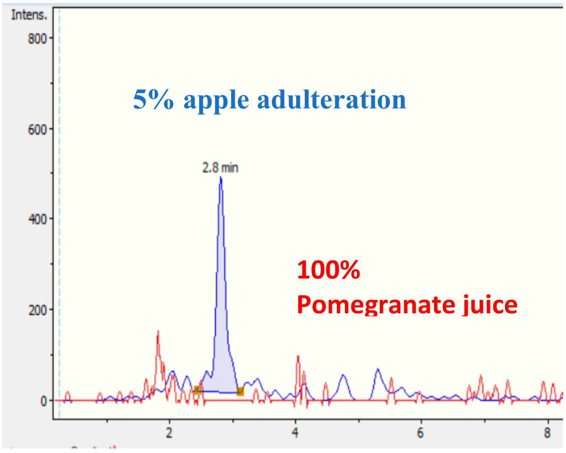

Fig S11d. EIC of m/z 289.0830 in authentic and adulterated pomegranate juice samples

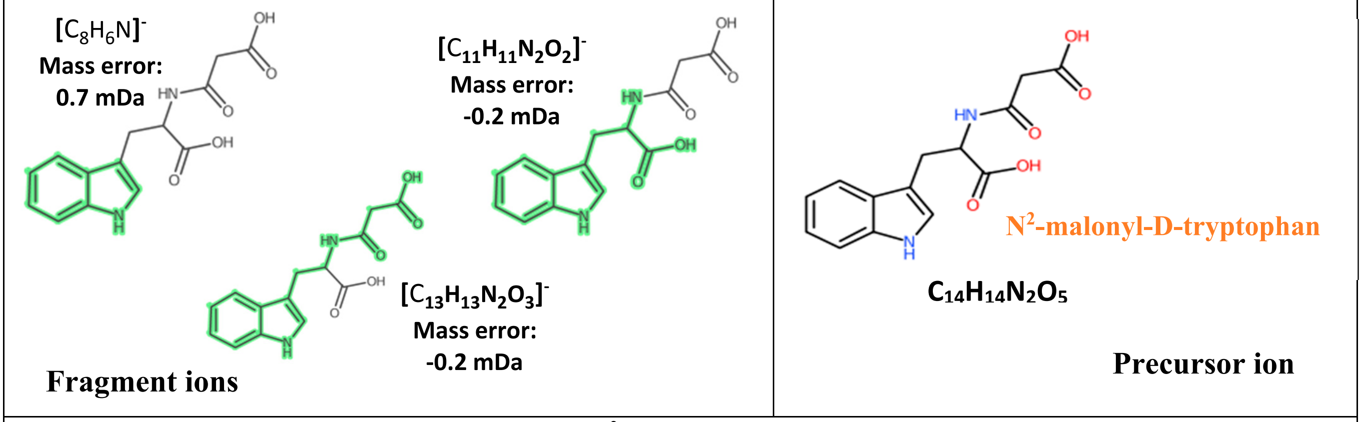

Fig S11e. Structures of precursor and fragment ions of N^{2}-malonyl-D-tryptophan

Figure S11. Identification data for the mass feature **m/z 289.0830 _2.8 min** (N^{2}-malonyl-D-tryptophan).

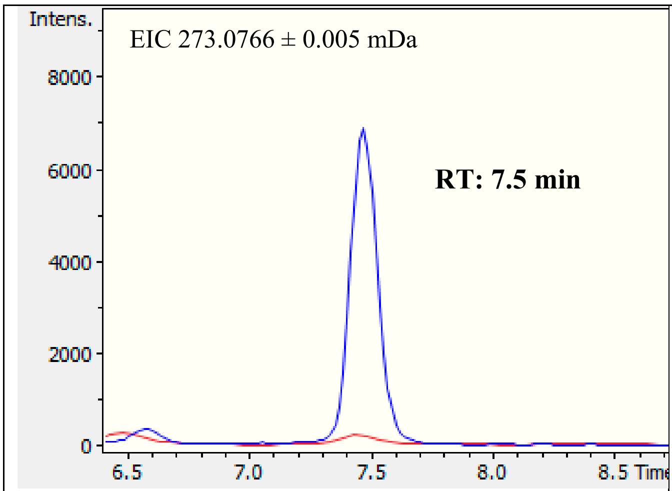

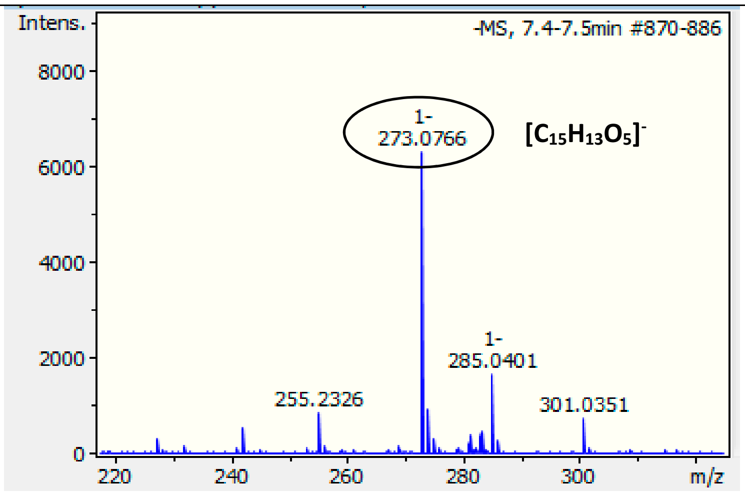

Fig S12a. EIC of m/z 273.0766 in pomegranate - apple juice

Fig S12b. MS Spectra of mass feature **273.0766_7.5 min**

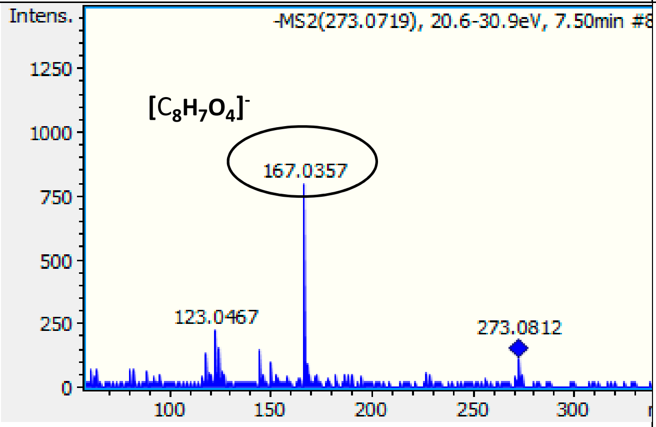

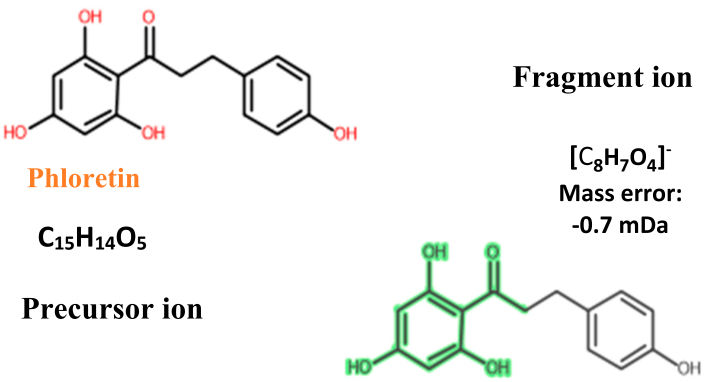

Fig S12c. MS//MS Spectra of mass feature **273.0766_7.5 min**

Fig S12d. Structures of precursor and fragment ions of phloretin

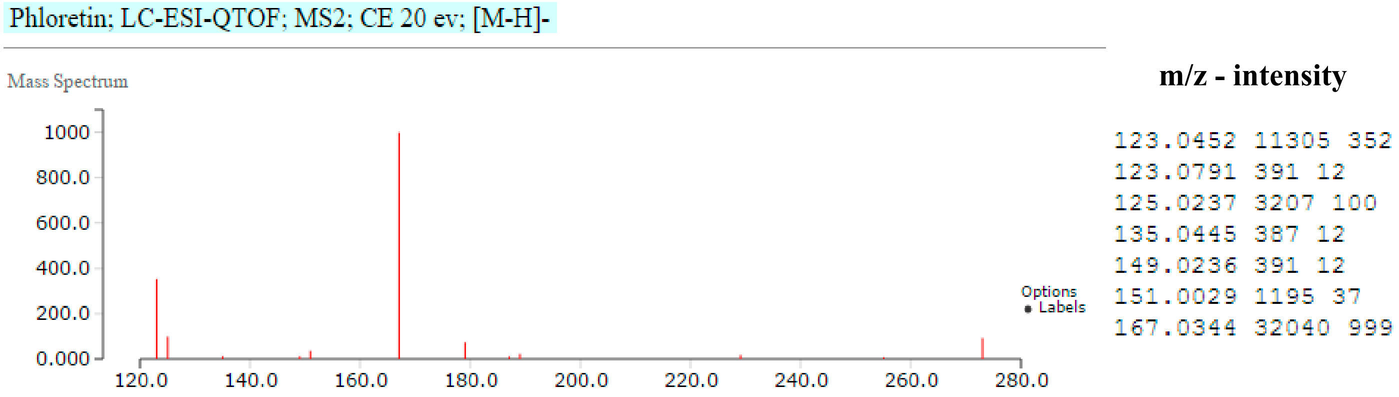

Fig. S12e: Mass Bank Record PB000702 (phloretin)

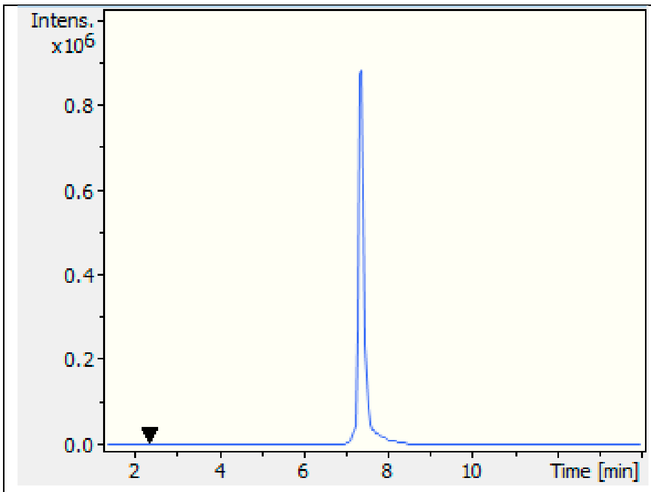

Fig. S12f: EIC of m/z 273.0766 in phloretin reference standard

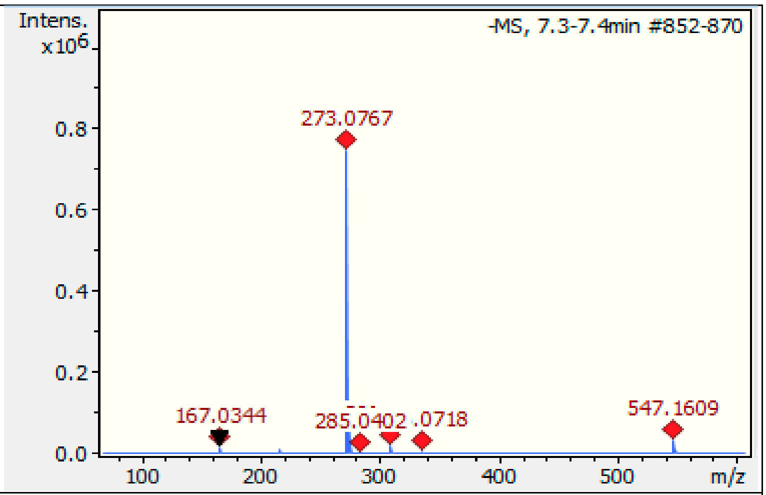

Fig. S12g: MS spectra of phloretin in reference standard

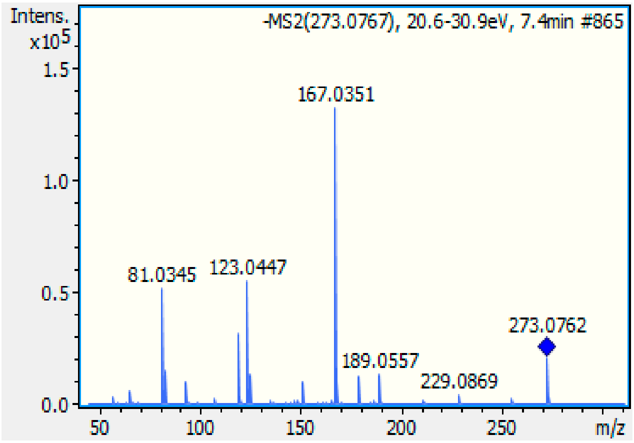

Fig. S12h: MS/MS spectra of phloretin in reference standard

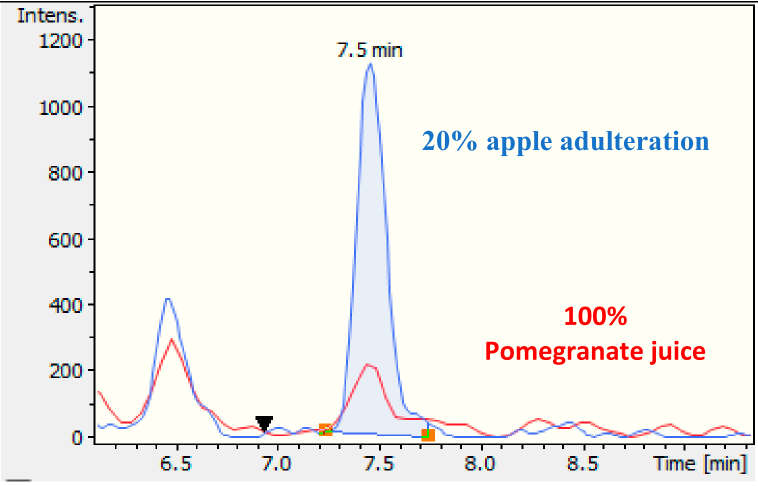

Fig. S12i: Phloretin in authentic and adulterated pomegranate juice samples

Figure S12. Identification data for the mass feature **m/z 273.0766 _7.5 min (**phloretin).

Tentative Identification of characteristic markers of grape juice obtained from untargeted workflow

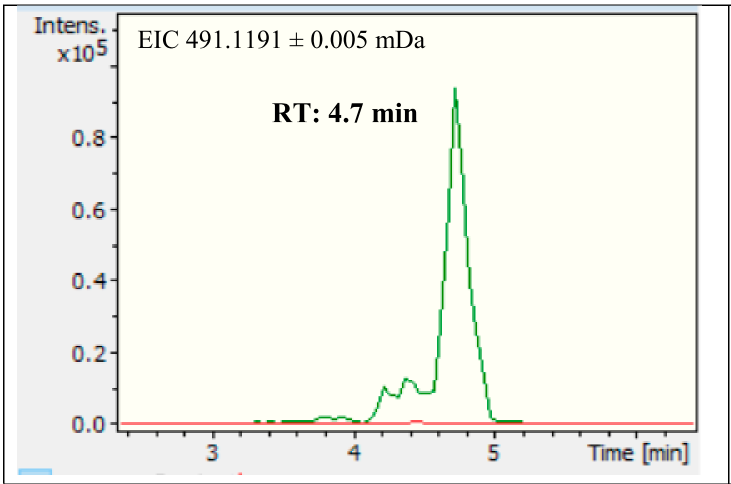

Fig S13a. EIC of m/z 491.1191 in pomegranate – grape juice

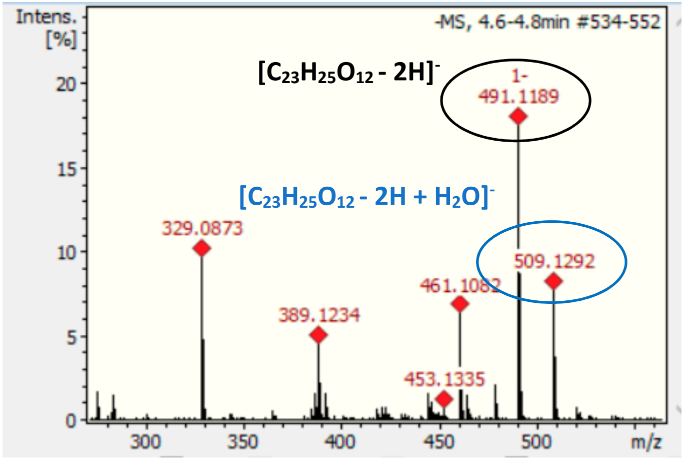

Fig S13b. MS Spectra of mass feature **m/z 491.1191_4.7 min**

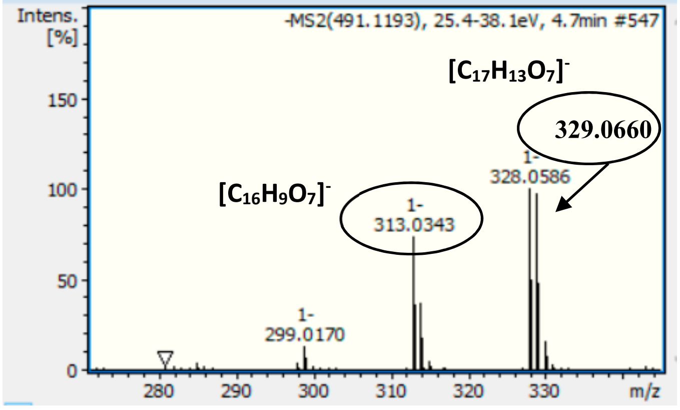

Fig S13c. MS/MS Spectra of mass feature **m/z 491.1191_4.7 min**

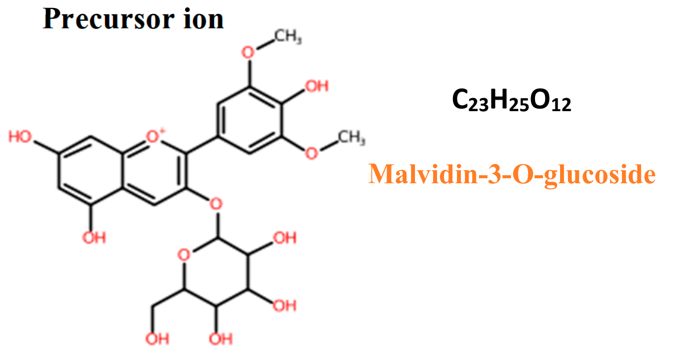

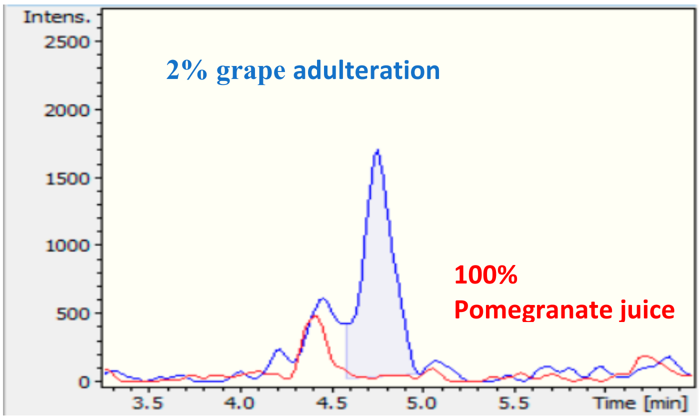

Fig S13d. EIC of m/z 491.1191 in authentic and adulterated pomegranate juice samples

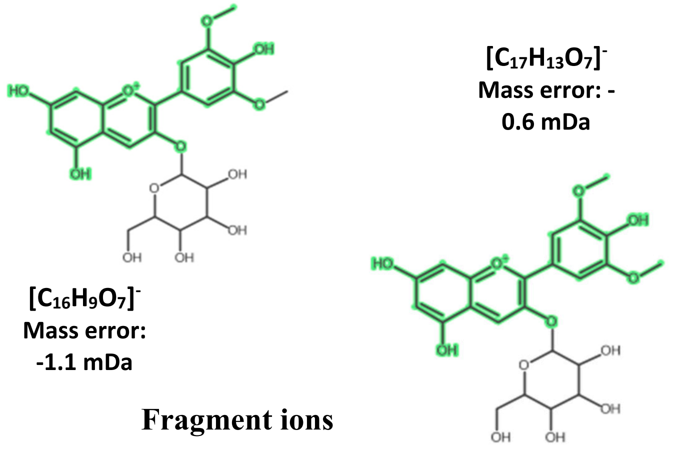

Fig S13e. Structures of precursor and fragment ions of malvidin-3-O-glucoside

Figure S13. Identification data for the mass feature **m/z 491.1191_4.7 min** (malvidin-3-O-glucoside).

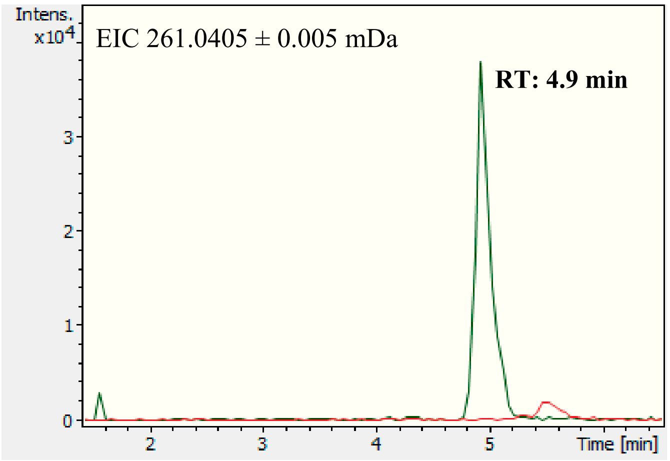

Fig S14a. EIC of m/z 261.0405 in pomegranate – grape juice

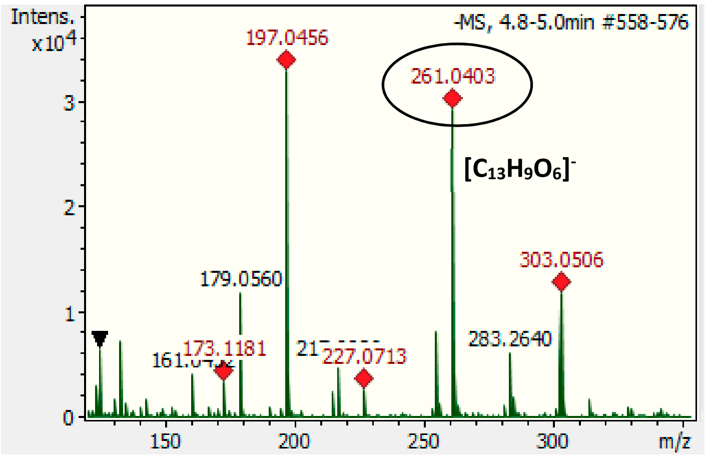

Fig S14b. MS Spectra of mass feature **m/z 261.0405_4.9 min**

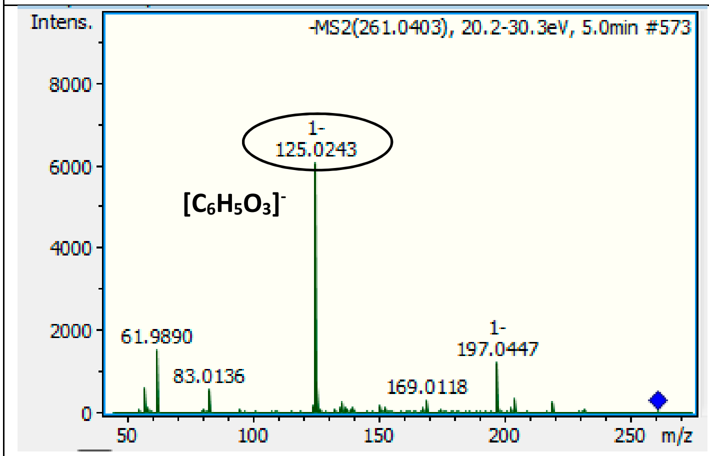

Fig S14c. MS/MS Spectra of mass feature **m/z 261.0405_4.9 min**

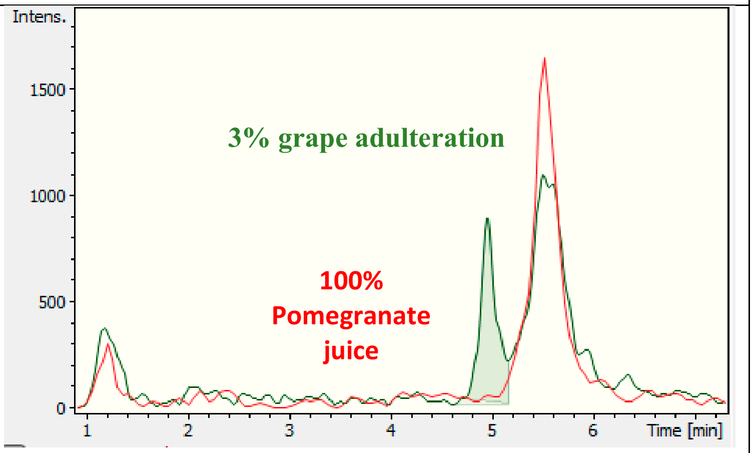

Fig S14d. EIC of m/z 261.0405 in authentic and adulterated pomegranate juice samples

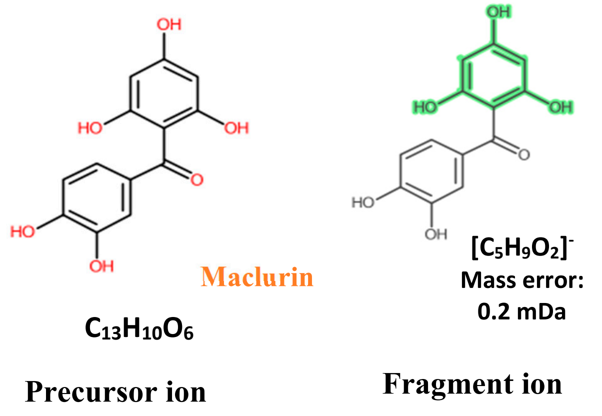

Fig S14e. Structures of precursor and fragment ions of maclurin

Figure S14. Identification data for the mass feature **m/z 261.0403 _4.9 min (**maclurin).

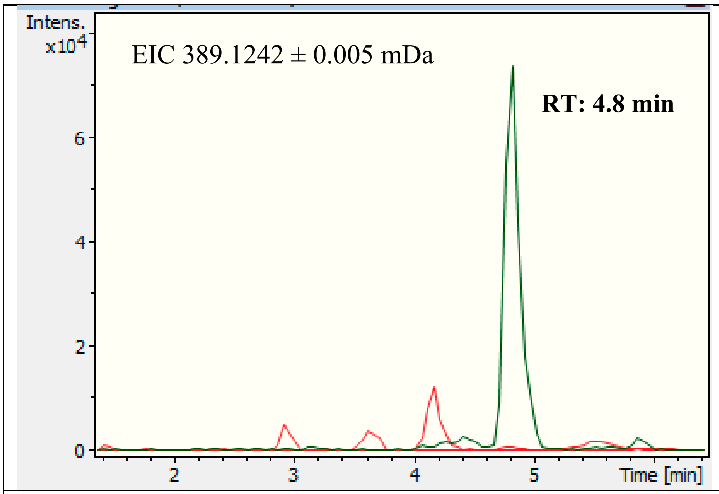

Fig S15a. EIC of m/z 389.1242 in pomegranate – grape juice

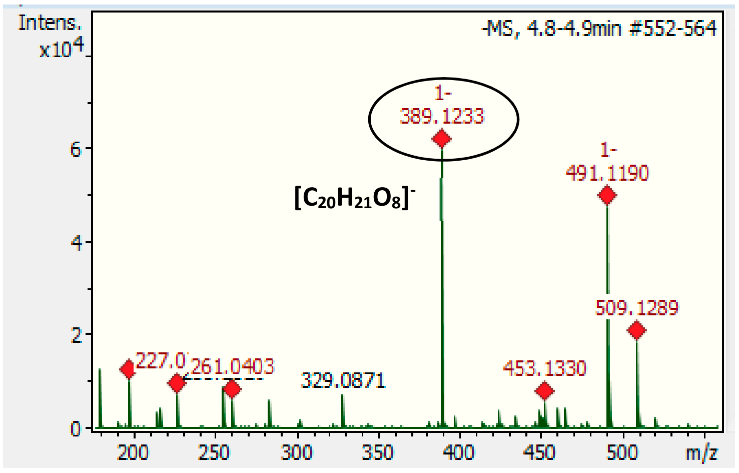

Fig S15b. MS Spectra of mass feature **m/z 389.1242_4.8 min**

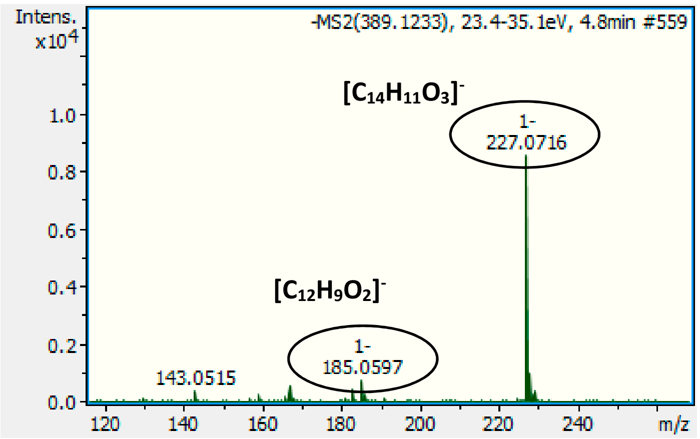

Fig S15c. MS/MS Spectra of mass feature **m/z 389.1242_4.8 min**

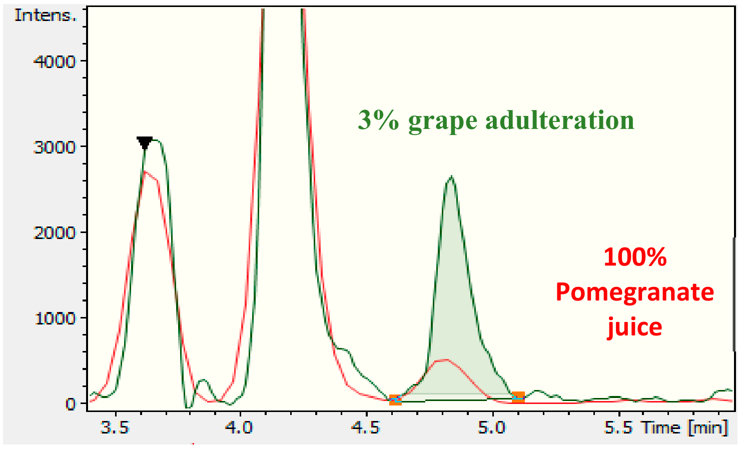

Fig S15d. EIC of m/z 389.1242 in authentic and adulterated pomegranate juice samples

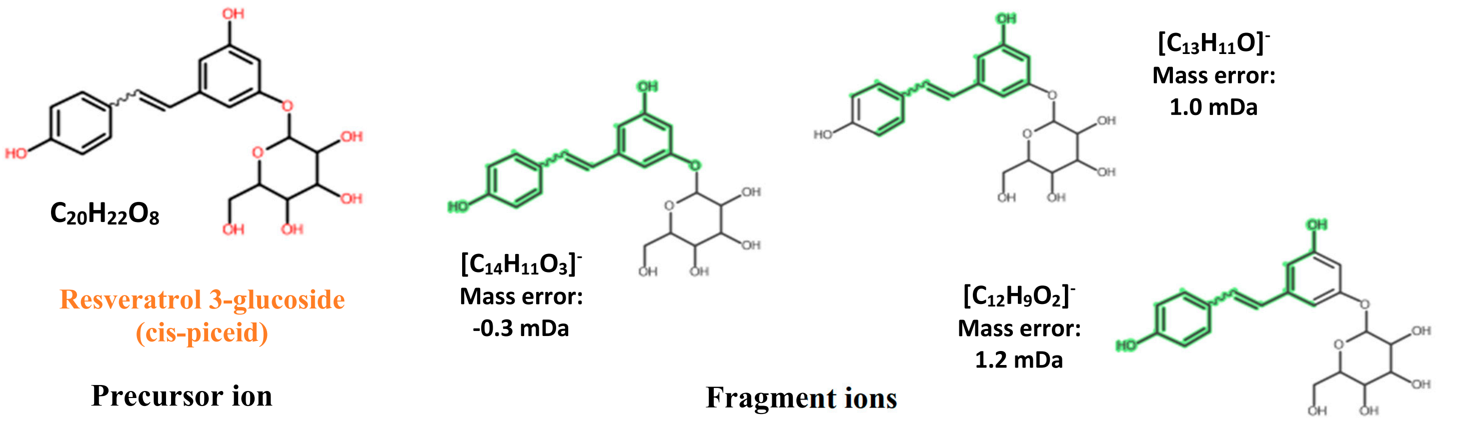

Fig S15e. Structures of precursor and fragment ions of resveratrol 3-glucoside (cis-piceid)

Figure S15. Identification data for the mass feature **m/z 389.1242 _4.8 min (**resveratrol 3-glucoside).

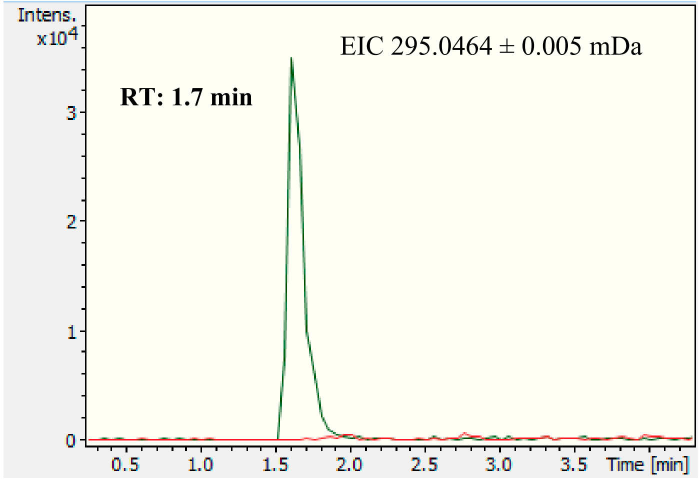

Fig S16a. EIC of m/z 295.0464 in pomegranate – grape juice

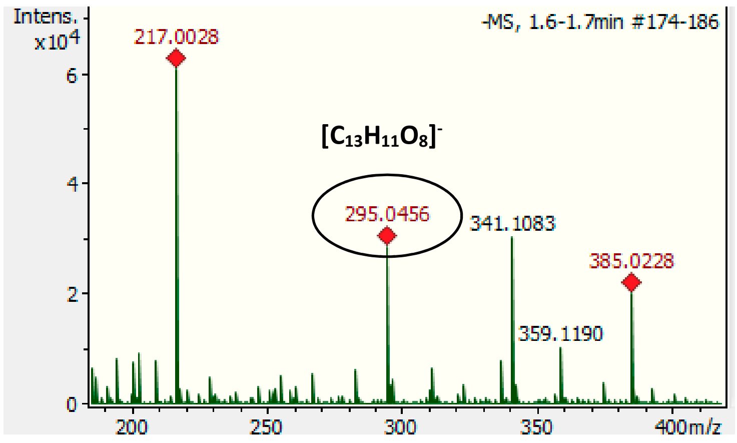

Fig S16b. MS Spectra of mass feature **m/z 295.0464_1.7 min**

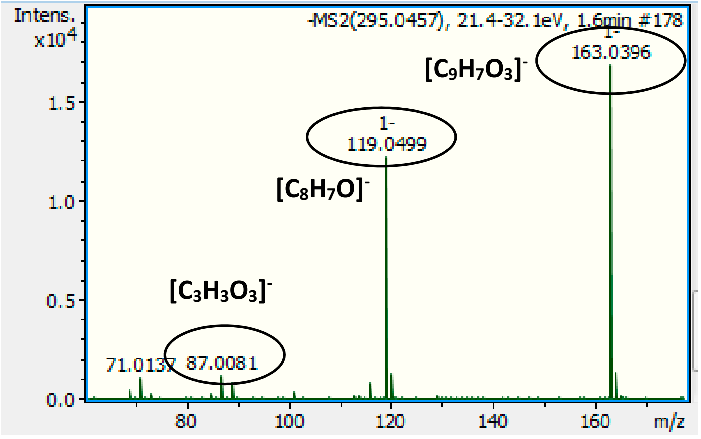

Fig S16c. MS/MS Spectra of mass feature **m/z 295.0464_1.7 min**

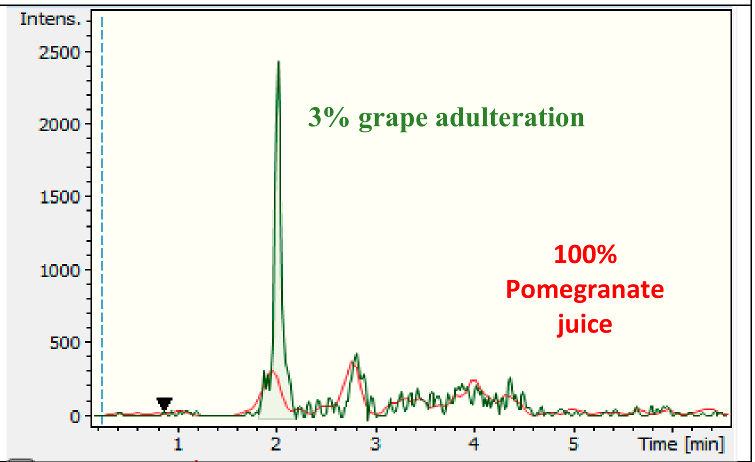

Fig S16d. EIC of m/z 295.0464 in authentic and adulterated pomegranate juice samples

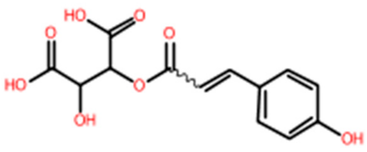

cis-coutaric acid

$C_{13}H_{12}O_8$

Precursor ion

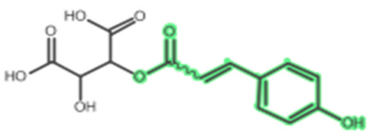

$[C_9H_7O_3]^-$
Mass error:
0.4 mDa

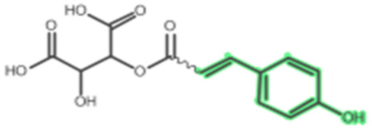

$[C_8H_7O]^-$
Mass error:
0.3 mDa

$[C_3H_3O_3]^-$
Mass error:
0.7 mDa

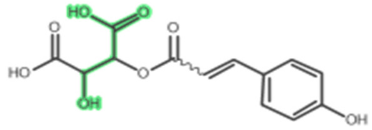

Fragment ions

Fig S16e. Structures of precursor and fragment ions of cis-coutaric acid

Figure S16. Identification data for the mass feature **m/z 295.0464_1.7 min** (cis-coutaric acid).

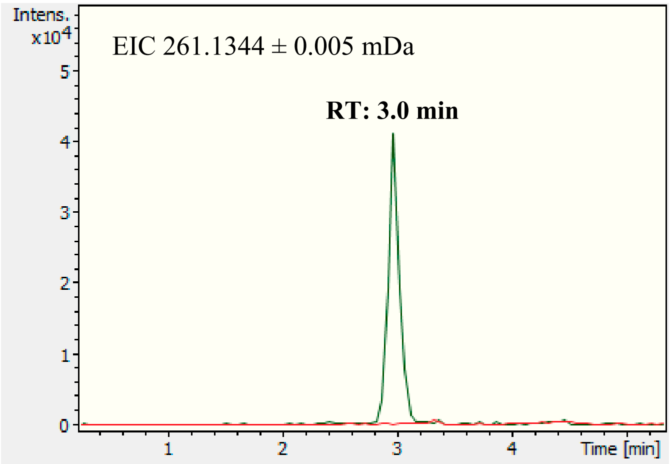

Fig S17a. EIC of m/z 261.1344 in pomegranate – grape juice

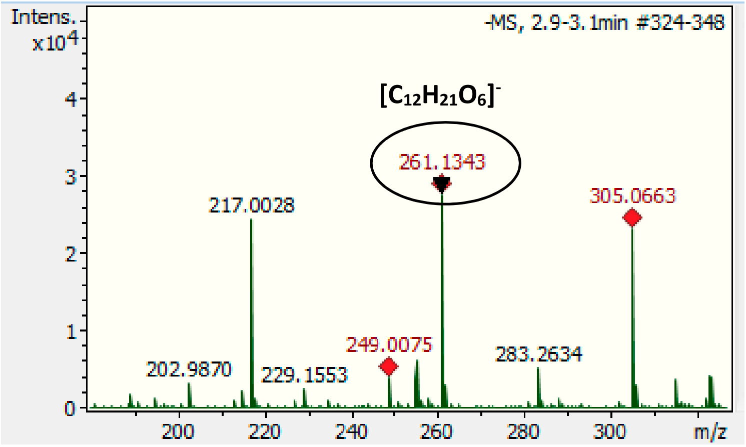

Fig S17b. MS Spectra of mass feature **m/z 261.1344_3.0 min**

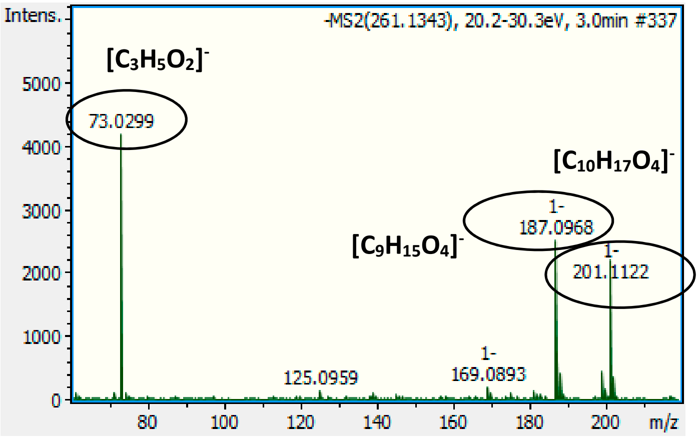

Fig S17c. MS/MS Spectra of mass feature **m/z 261.1344_3.0 min**

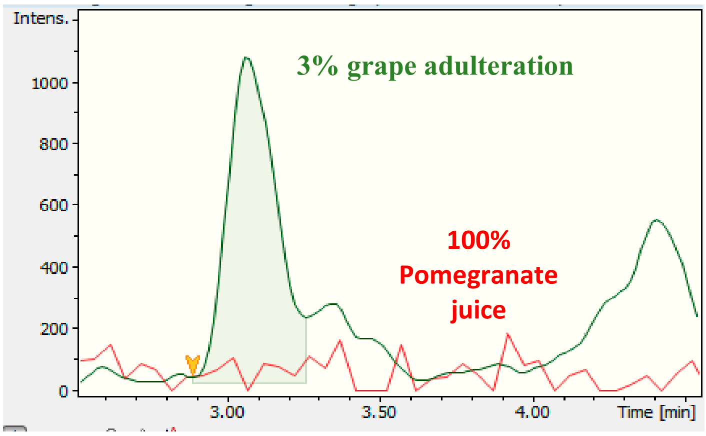

Fig S17d. EIC of m/z 261.1344 in authentic and adulterated pomegranate juice samples

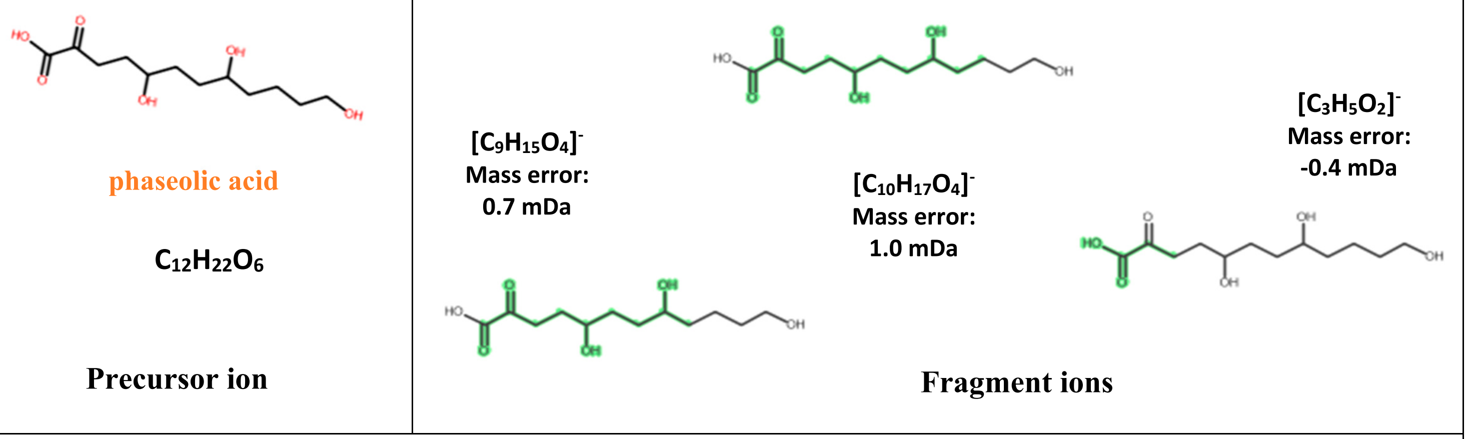

Fig S17e. Structures of precursor and fragment ions of phaseolic acid

Figure S17. Identification data for the mass feature **m/z 261.1344_3.0 min** (phaseolic acid).

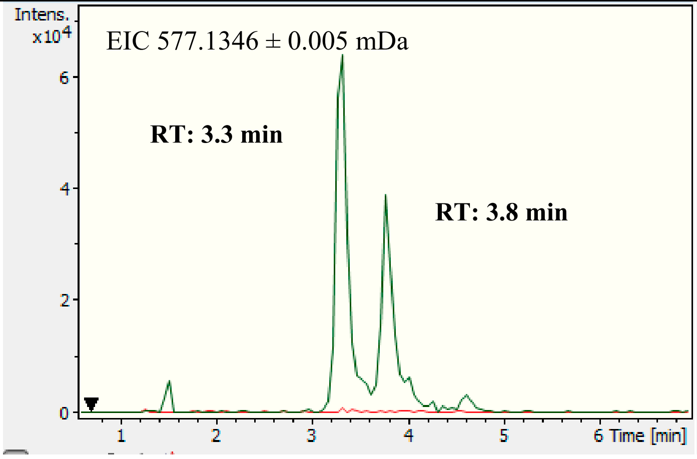

Fig S18a. EIC of m/z 577.1346 in **pomegranate – grape juice**

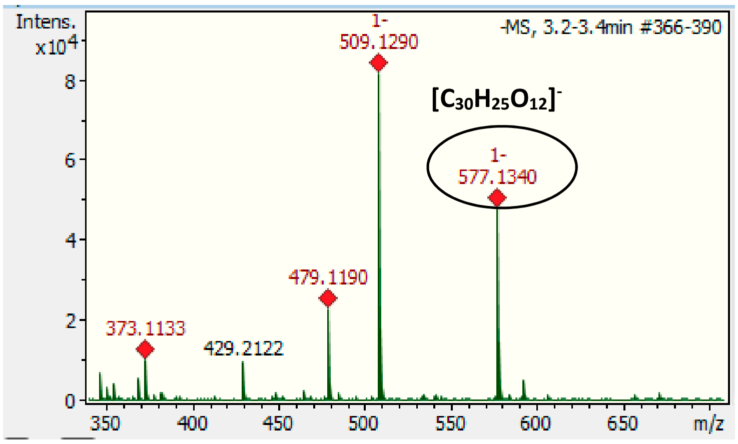

Fig S18b. MS Spectra of mass feature **m/z 577.1346_3.3 min**

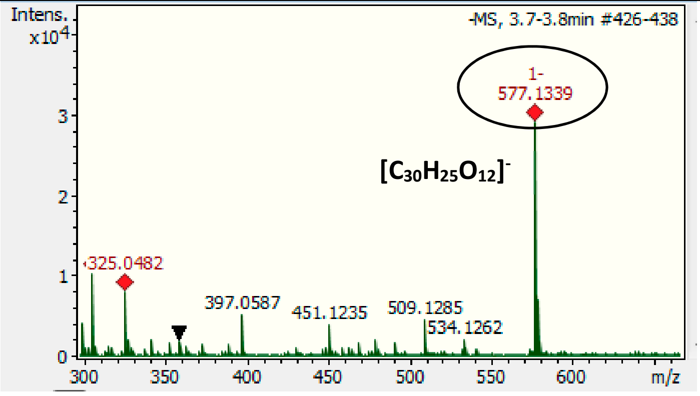

Fig S18c. MS Spectra of mass feature **m/z 577.1346_3.8 min**

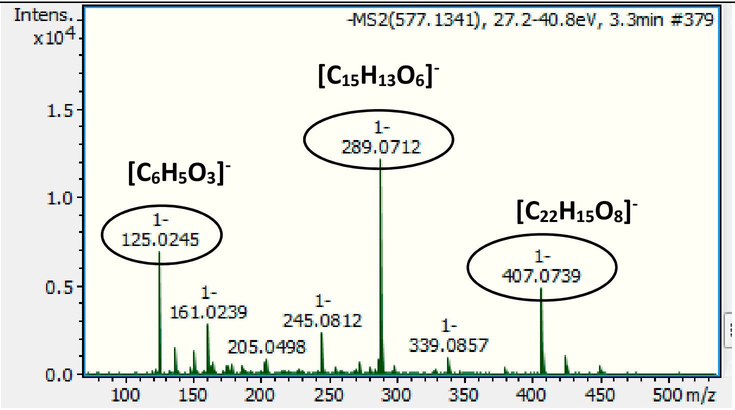

Fig S18d. MS/MS Spectra of mass feature **m/z 577.1346_3.3 min**

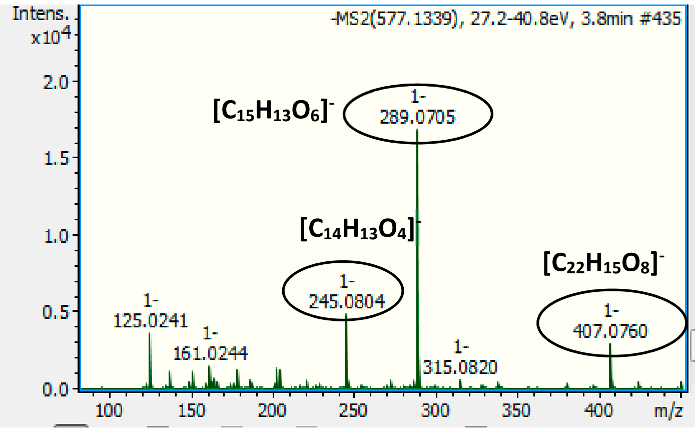

Fig S18e. MS/MS Spectra of mass feature **m/z 577.1346_3.8 min**

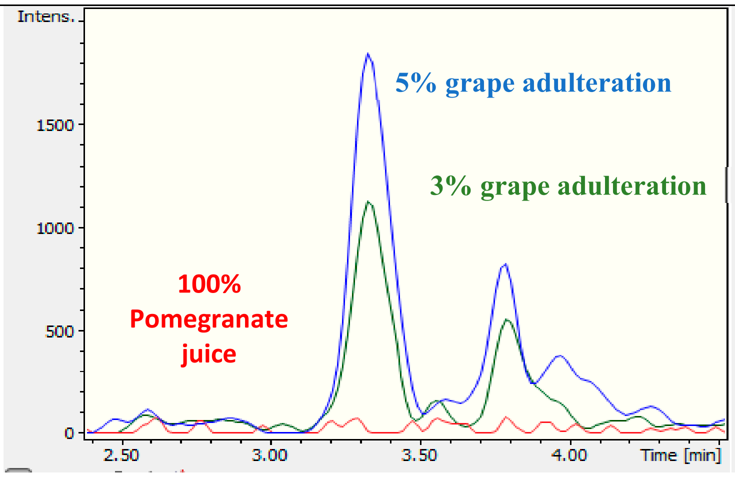

Fig S18f. EIC of m/z 577.1346 in authentic and adulterated pomegranate juice samples

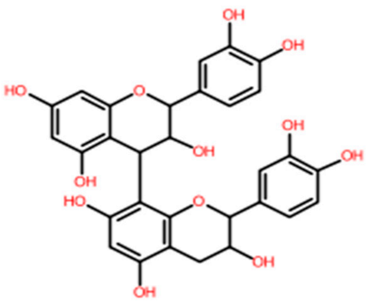

$C_{30}H_{26}O_{12}$

Procyanidin B isomer

Precursor ion

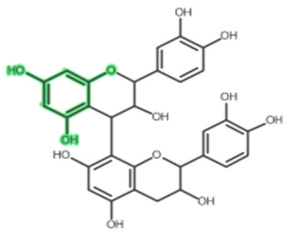

$[C_6H_5O_3]^-$
Mass error:
-0.1 mDa

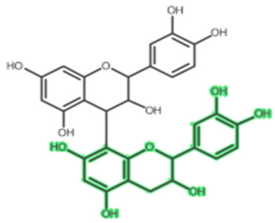

$[C_{15}H_{13}O_6]^-$
Mass error:
0.5 mDa

$[C_{14}H_{13}O_4]^-$
Mass error:
0.8 mDa

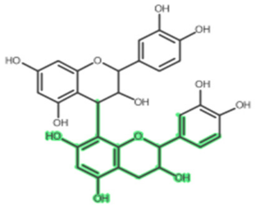

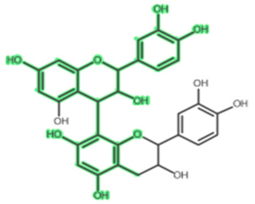

$[C_{22}H_{15}O_8]^-$
Mass error:
1.2 mDa

Fragment ions

Fig S18e. Structures of precursor and fragment ions of procyanidin B isomer

Figure S18. Identification data for the mass feature **m/z 261.1344_3.0 min** (procyanidin B).
